# Supplementary material for: Residential green space, air pollution, and related metabolites in association with depression among cancer survivors
Source: Nat Commun. 2026 Mar 9;17:3690. doi: 10.1038/s41467-026-70393-4 (PMC13100056; doi:10.1038/s41467-026-70393-4)
Supplement: Supplementary file 1 — Supplementary Information [file 41467_2026_70393_MOESM1_ESM.pdf]

Supplementary Materials

Residential Green Space, Air Pollution, and Related Metabolites in Association with Depression Among Cancer Survivors

Jianhui Zhao[1,2*], Jingyu Ye[1*], Erxu Xue[1,3*], Liying Xu[1], Jing Sun[1], Siyun Zhou[1], Tengfei Li[1,4], Haoze Cao[1,4], Zhongquan Sun[4], Weilin Wang[4], Yazhou He[5], Yuan Ding[4,6,7#], Xue Li[1,8,9#]

[1] Department of Big Data in Health Science, The Second Affiliated Hospital, School of Public Health, Zhejiang University School of Medicine, Hangzhou, Zhejiang, China

[2] Clinical and Translational Epidemiology Unit, Massachusetts General Hospital and Harvard Medical School, Boston, MA, USA

[3] The D. H. Chen School of Universal Health, Sir Run Run Shaw Hospital, Zhejiang University School of Medicine, Hangzhou, Zhejiang, China

[4] Department of Hepatobiliary and Pancreatic Surgery, The Second Affiliated Hospital, Zhejiang University School of Medicine, Hangzhou, Zhejiang, China

[5] Department of Oncology, West China School of Public Health and West China Fourth Hospital, Sichuan University, Chengdu, Sichuan, China

[6] Key Laboratory of Precision Diagnosis and Treatment for Hepatobiliary and Pancreatic Tumor of Zhejiang Province, Hangzhou, Zhejiang, China

[7] Cancer Center, Zhejiang University, Hangzhou, Zhejiang, China

[8] Zhejiang Key Laboratory of Intelligent Preventive Medicine, Hangzhou, Zhejiang, China

[9] Centre for Global Health, Usher Institute, University of Edinburgh, Edinburgh, United Kingdom

* These authors contributed equally: Jianhui Zhao, Jingyu Ye, Erxu Xue.

Correspondence to: Xue Li, Email: xueli157@zju.edu.cn, OCIRD: 0000-0001-6880-2577; Yuan Ding, Email: dingyuan@zju.edu.cn.

Supplementary Method

Supplementary Fig. 1. Restricted cubic spline curves of green space (A) and natural environment (B) at 1000 m buffer and incident depression risk among cancer survivors (N=21,507).

Supplementary Fig. 2. Mediation analysis of air pollutants and air pollution score in the association between green space, blue space, natural environment at 300 m buffer and depression risk (N=21,507).

Supplementary Fig. 3. Mediation analysis of air pollutants and air pollution score in the association between green space, blue space, natural environment at 1000 m buffer and depression risk (N=21,507).

Supplementary Fig. 4. The metabolites' coefficients (weights) in the green space- (A) and natural environment- (B) related metabolic signatures and associations of the 45 (C) and 58 (D) selected metabolites with nature exposures and air pollutants.

Supplementary Fig. 5. The metabolites' coefficients (weights) in the APS-related metabolic signature (A) and associations of the 61 selected metabolites with nature exposures and air pollutants (B).

Supplementary Fig. 6. The association between green space-related metabolites and depression among cancer survivors (N=21,507).

Supplementary Fig. 7. The association between natural environment-related metabolites and depression among cancer survivors (N=21,507).

Supplementary Fig. 8. The association between air pollution score-related metabolites and depression among cancer survivors (N=21,507).

Supplementary Fig. 9. Associations between exposure to green space, blue space and natural environment at 300 m buffer and depression risk in stratified analyses.

Supplementary Fig. 10. Associations between exposure to green space, blue space and natural environment at 1000 m buffer and depression risk in stratified analyses.

Supplementary Fig. 11. Associations between air pollutants and depression risk in stratified analyses.

Supplementary Fig. 12. Flow chart of selection of study participants.

Supplementary Fig. 13. Distribution of cancer types and survival time among cancer survivors (N=21,507).

Supplementary Fig. 14. Distribution of cancer types and diagnosis-to-enrollment interval among cancer survivors (N=21,507).

Supplementary Fig. 15. Distribution of cancer survivors across levels of green space, blue space, and natural environment exposure within 300 m (A, B and C) and 1000 m (D, E and F) buffers (N=21,507).

Supplementary Fig. 16. A brief schematic of β in the AFT model and HR in the Cox model interpretation.

Supplementary Table 1. Distribution of residential green space, blue space and natural environment.

Supplementary Table 2. Correlation matrix of environmental exposures.

Supplementary Table 3. Associations between green space, blue space, and natural environment at 300 m buffer and the risk of depression among cancer survivors (N=21,507).

Supplementary Table 4. Associations between green space, blue space, and natural environment at 1000 m buffer and the risk of depression among cancer survivors (N=21,507).

Supplementary Table 5. Mediation analysis of air pollutants and air pollution score in the association between green space, blue space, natural environment at 300 m buffer and depression risk (N=21,507).

Supplementary Table 6. Mediation analysis of air pollutants and air pollution score in the association between green space, blue space, natural environment at 1000 m buffer and depression risk (N=21,507).

Supplementary Table 7. Associations between green space-related metabolic signature and green space and air pollutants.

Supplementary Table 8. Associations between nature environment-related metabolic signature and nature environment and air pollutants.

Supplementary Table 9. Associations between air pollution score-related metabolic signature and green space, nature environment, and air pollutants.

Supplementary Table 10. Associations between environmental exposures at 300 m buffer and depression risk in breast cancer patients (N=7,365).

Supplementary Table 11. Associations between environmental exposures at 1000 m buffer and depression risk in breast cancer patients (N=7,365).

Supplementary Table 12. Associations between environmental exposures at 300 m buffer and depression risk in melanoma skin cancer patients (N=1,721).

Supplementary Table 13. Associations between environmental exposures at 1000 m buffer and depression risk in melanoma skin cancer patients (N=1,721).

Supplementary Table 14. Associations between environmental exposures at 300 m buffer and depression risk in non-melanoma skin cancer patients (N=14,896).

Supplementary Table 15. Associations between environmental exposures at 1000 m buffer and depression risk in non-melanoma skin cancer patients (N=14,896).

Supplementary Table 16. Associations between environmental exposures at 300 m buffer and depression risk in lung cancer patients (N=265).

Supplementary Table 17. Associations between environmental exposures at 1000 m buffer and depression risk in lung cancer patients (N=265).

Supplementary Table 18. Associations between environmental exposures at 300 m buffer and depression risk in prostate cancer patients (N=2,547).

Supplementary Table 19. Associations between environmental exposures at 1000 m buffer and depression risk in prostate cancer patients (N=2,547).

Supplementary Table 20. Associations between environmental exposures at 300 m buffer and depression risk in colorectal cancer patients (N=1,897).

Supplementary Table 21. Associations between environmental exposures at 1000 m buffer and depression risk in colorectal cancer patients (N=1,897).

Supplementary Table 22. Associations between environmental exposures at 300 m buffer and risk of depression after adjusting separately for air pollutants (N=21,507).

Supplementary Table 23. Associations between environmental exposures at 1000 m buffer and risk of depression after adjusting separately for air pollutants (N=21,507).

Supplementary Table 24. Associations between environmental exposures at 300 m and 1000 m buffers and risk of depression after adjusting separately for all air pollutants and air pollution score (N=21,507).

Supplementary Table 25. Associations between environmental exposures at 300 m and 1000 m buffers and risk of depression after adjusting separately for cancer type and sleep pattern (N=21,507).

Supplementary Table 26. Associations between environmental exposures at 300 m buffer and the risk of depression after removing antidepressant use from the covariate set (N=21,507), excluding those diagnosed with depression within 1 year (N=21,437) or 3 years (N=21,279) post-cancer, and those who died within 10 years after cancer diagnosis (N=20,522).

Supplementary Table 27. Associations between environmental exposures at 1000 m buffer and the risk of depression after removing antidepressant use from the covariate set (N=21,507), excluding those diagnosed with depression within 1 year (N=21,437) or 3 years (N=21,279) post-cancer, and those who died within 10 years after cancer diagnosis (N=20,522).

Supplementary Table 28. Associations between environmental exposures at 300 m buffer and the risk of depression, restricted to individuals who had lived at their current address for more than 10 years before baseline (N=15,162).

Supplementary Table 29. Associations between environmental exposures at the 1000 m buffer and the risk of depression, restricted to individuals who had lived at their current address for more than 10 years before baseline (N=15,162).

Supplementary References

Supplementary Method

Quality control and batch effect adjustment applied during nuclear magnetic resonance (NMR) metabolomics measurements in the UK Biobank

The UK Biobank and Nightingale Health implemented a series of rigorous quality control (QC) procedures to minimize batch effect–related biases, which are known to significantly affect metabolomics profiling (https://biobank.ndph.ox.ac.uk/ukb/label.cgi?id=220). First, consensus was reached on predefined quality metrics to ensure consistency across samples, and pilot measurements were conducted. Details of the Nightingale Health NMR biomarker platform have been described previously [1,2]. The NMR platform maintained consistent results across different spectrometers and over time, while requiring minimal sample preparation (no extraction steps), which contributed to high reproducibility. During measurement of UK Biobank samples, Nightingale Health continuously monitored within- and between-spectrometer consistency. Each 96-well plate included two internal control samples (Nightingale Health) and two blind duplicate samples (UK Biobank). Predefined coefficients of variation (CVs) were applied across the biomarker spectrum, with most biomarkers achieving CV <5%. Biomarkers strongly influenced by interfering substances were excluded. This platform has been recognized for its long-term reproducibility and absence of major batch effects, enabling metabolomics data to be analyzed in the same way as clinical biochemistry data in the UK Biobank.

To further improve data utility, the UK Biobank identified technical sources of variation such as sample preparation time, shipping plate well, spectrometer batch, temporal drift, and outlier plates [3]. A statistical procedure was then developed to mitigate these effects and shown to enhance genetic and epidemiological signals. Additional QC procedures were made publicly available via the R package ukbnmr, enabling removal of residual technical variation from biomarker concentrations. Although most biomarkers proved robust, ~22 of the 249 biomarkers showed ≥5% of variance explained by technical factors, most notably spectrometer differences and within-spectrometer temporal drift. To address this, median normalization at the plate level was suggested, although this could also remove biological variation due to the limited number of samples (94 per plate). In addition, a log transformation was applied to biomarker concentrations to reduce the impact of potential sample degradation.

Individual metabolite concentrations were natural log-transformed (ln[x+1]) and standardized to z-scores prior to analysis.

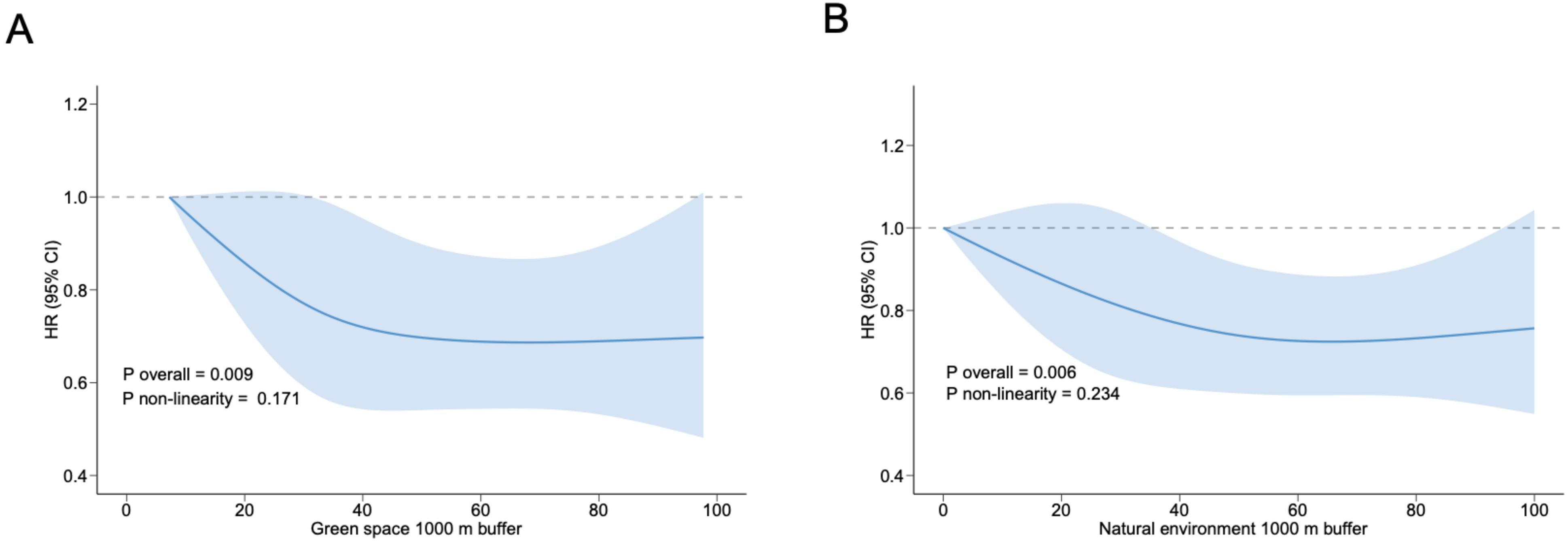

Supplementary Fig. 1. Restricted cubic spline curves of green space (A) and natural environment (B) at 1000 m buffer and incident depression risk among cancer survivors (N=21,507).

HRs and 95% CIs were estimated using Cox proportional hazards regression with restricted cubic spline functions to model potential non-linear associations, with adjustment for age, sex and ethnicity, educational level, household income, employment status, body mass index, smoking status, drinking status, physical activity, diet and antidepressant use. P values for overall association and non-linearity were obtained from Wald tests. All P values were based on two-sided tests. CI confidence interval, HR hazard ratio.

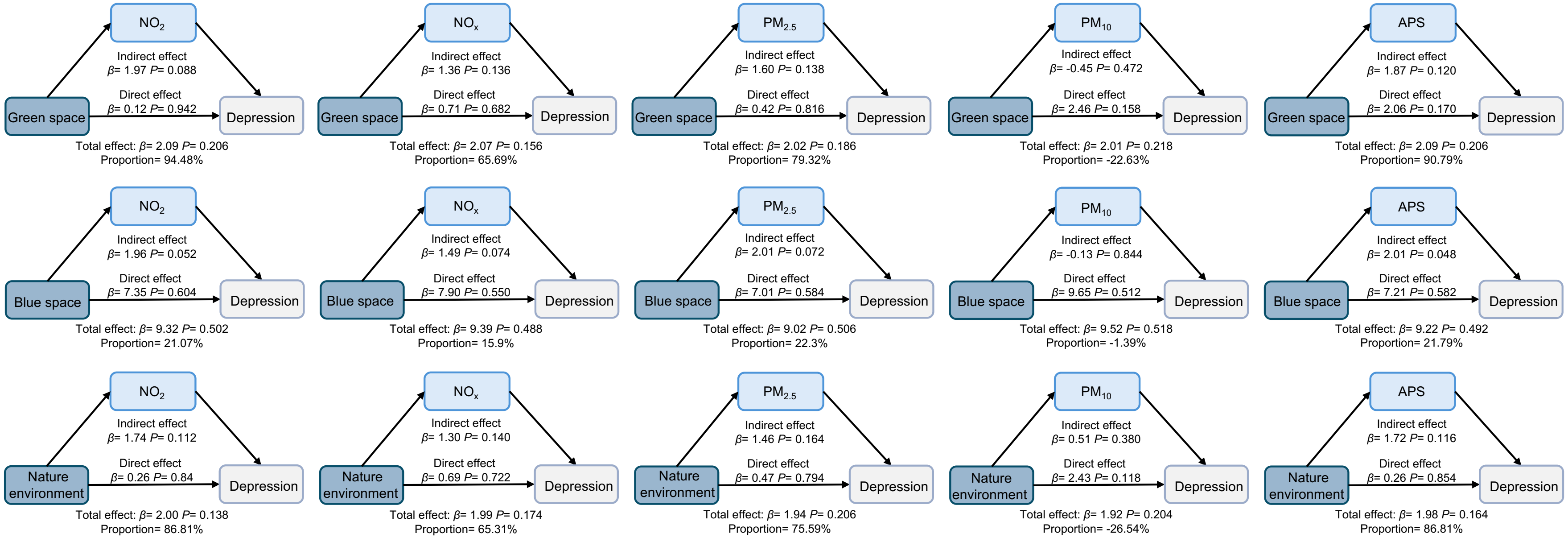

Supplementary Fig. 2. Mediation analysis of air pollutants and air pollution score in the association between green space, blue space, natural environment at 300 m buffer and depression risk (N=21,507).

This mediation analysis was performed using an accelerated failure time model with covariates adjusted as in Model 3, adjusted for age, sex and ethnicity, educational level, household income, employment status, body mass index, smoking status, drinking status, physical activity, diet and antidepressant use. The regression coefficient (β) represents the effect of exposure on log time to depression onset, where positive values indicate delayed onset (lower risk) and negative values indicate earlier onset (higher risk). The proportion mediated was quantified as the ratio of the mediation effect to the total effect (Detailed β, proportion, 95% CI, and P-value are summarized in Supplementary Table 5). All P values were based on two-sided tests. NO_2 nitrogen dioxide, NO_x nitrogen oxides, PM_{10} particulate matter (PM) with aerodynamic diameter ≤10 μm, $PM_{2.5}$ PM with aerodynamic diameter < 2.5 μm.

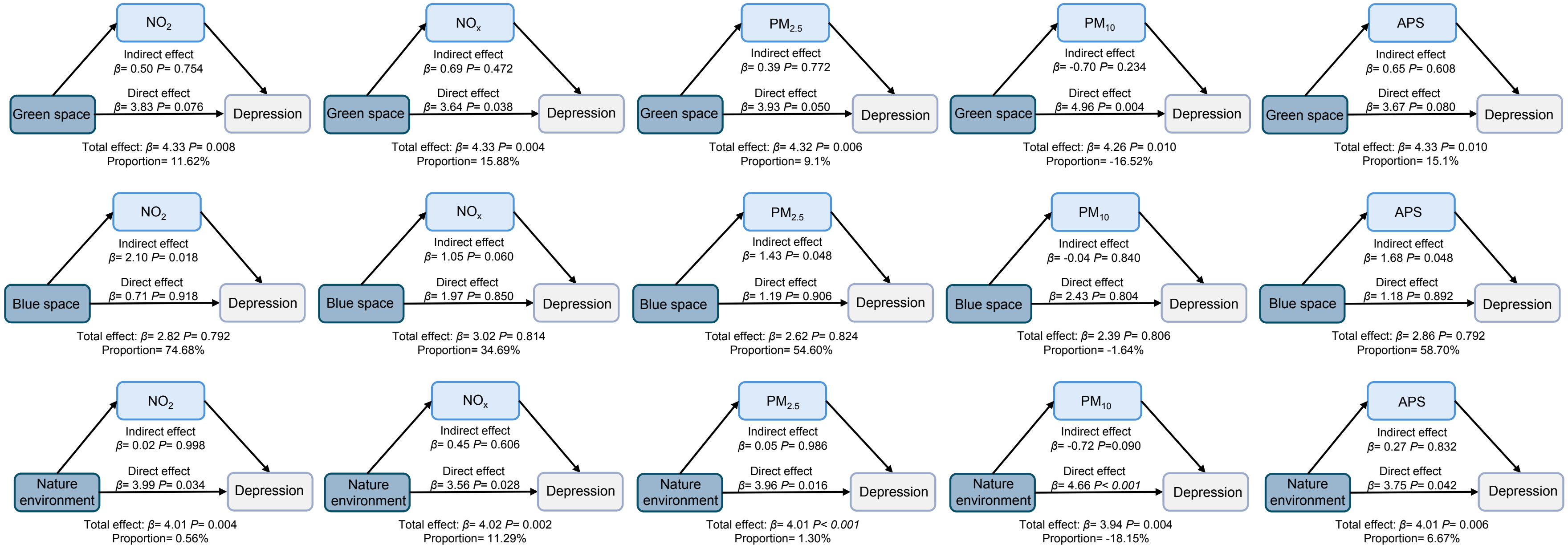

Supplementary Fig. 3. Mediation analysis of air pollutants and air pollution score in the association between green space, blue space, natural environment at 1000 m buffer and depression risk (N=21,507).

This mediation analysis was performed using an accelerated failure time model with covariates adjusted as in Model 3, adjusted for age, sex and ethnicity, educational level, household income, employment status, body mass index, smoking status, drinking status, physical activity, diet and antidepressant use. The regression coefficient (β) represents the effect of exposure on log time to depression onset, where positive values indicate delayed onset (lower risk) and negative values indicate earlier onset (higher risk). The proportion mediated was quantified as the ratio of the mediation effect to the total effect (Detailed β, proportion, 95% CI, and P-value are summarized in Supplementary Table 6). All P values were based on two-sided tests. NO_2 nitrogen dioxide, NO_x nitrogen oxides, PM_{10} particulate matter (PM) with aerodynamic diameter ≤10 μm, $PM_{2.5}$ PM with aerodynamic diameter < 2.5 μm.

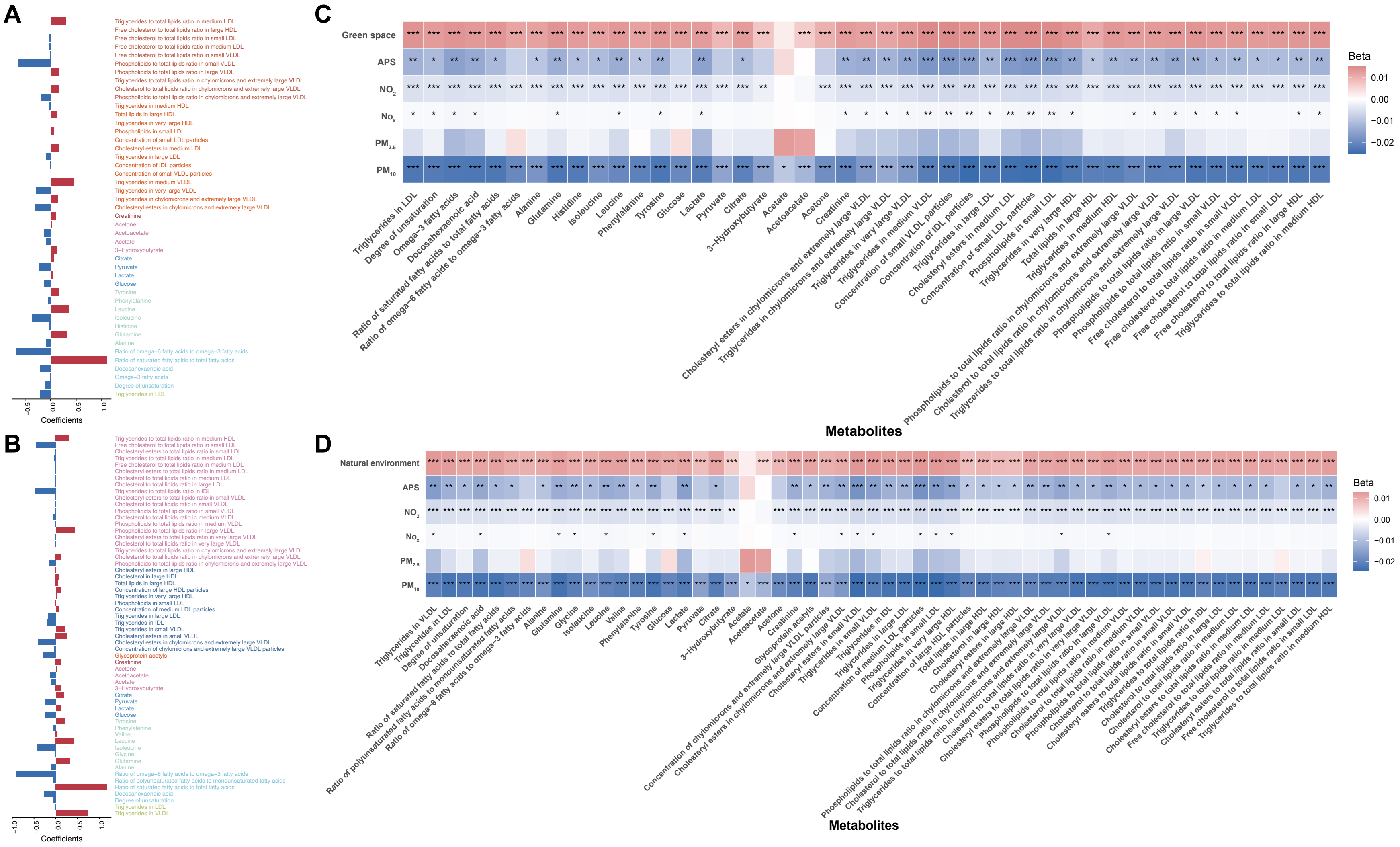

Supplementary Fig. 4. The metabolites' coefficients (weights) in the green space- (A) and natural environment- (B) related metabolic signatures and associations of the 45 (C) and 58 (D) selected metabolites with nature exposures and air pollutants.

Colors denote the association directions (red-positive and blue-inverse) and magnitudes (the darker the color, the stronger the magnitude); asterisks represent the significance of association (* FDR corrected P < 0.05; ** FDR corrected P < 0.01; *** FDR corrected P < 0.001). Multiple linear regression model was adjusted for age, sex and ethnicity, educational level, household income, employment status, body mass index, smoking status, drinking status, physical activity, diet and antidepressant use. Exact P values are provided in the Source Data file, all P values were two-sided, and the FDR method was applied to adjust for multiple comparisons. FDR False Discovery Rate, HDL high-density lipoprotein, LDL low-density lipoprotein, VLDL very LDL. Source data are provided with this publication as a Source Data file.

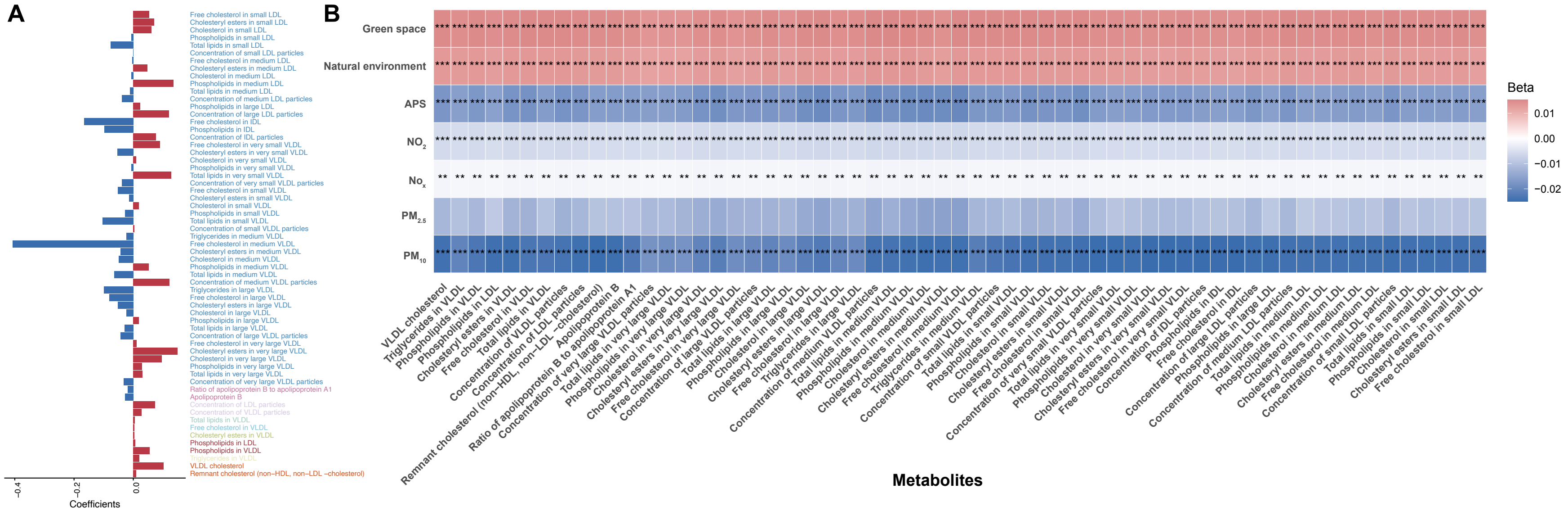

Supplementary Fig. 5. The metabolites' coefficients (weights) in the APS-related metabolic signature (A) and associations of the 61 selected metabolites with nature exposures and air pollutants (B).

Colors denote the association directions (red-positive and blue-inverse) and magnitudes (the darker the color, the stronger the magnitude); asterisks represent the significance of association (* FDR corrected P < 0.05; ** FDR corrected P < 0.01; *** FDR corrected P < 0.001). Multiple linear regression model was adjusted for age, sex and ethnicity, educational level, household income, employment status, body mass index, smoking status, drinking status, physical activity, diet and antidepressant use. Exact P values are provided in the Source Data file, all P values were two-sided, and the FDR method was applied to adjust for multiple comparisons. FDR False Discovery Rate, HDL high-density lipoprotein, LDL low-density lipoprotein, VLDL very LDL. Source data are provided with this publication as a Source Data file.

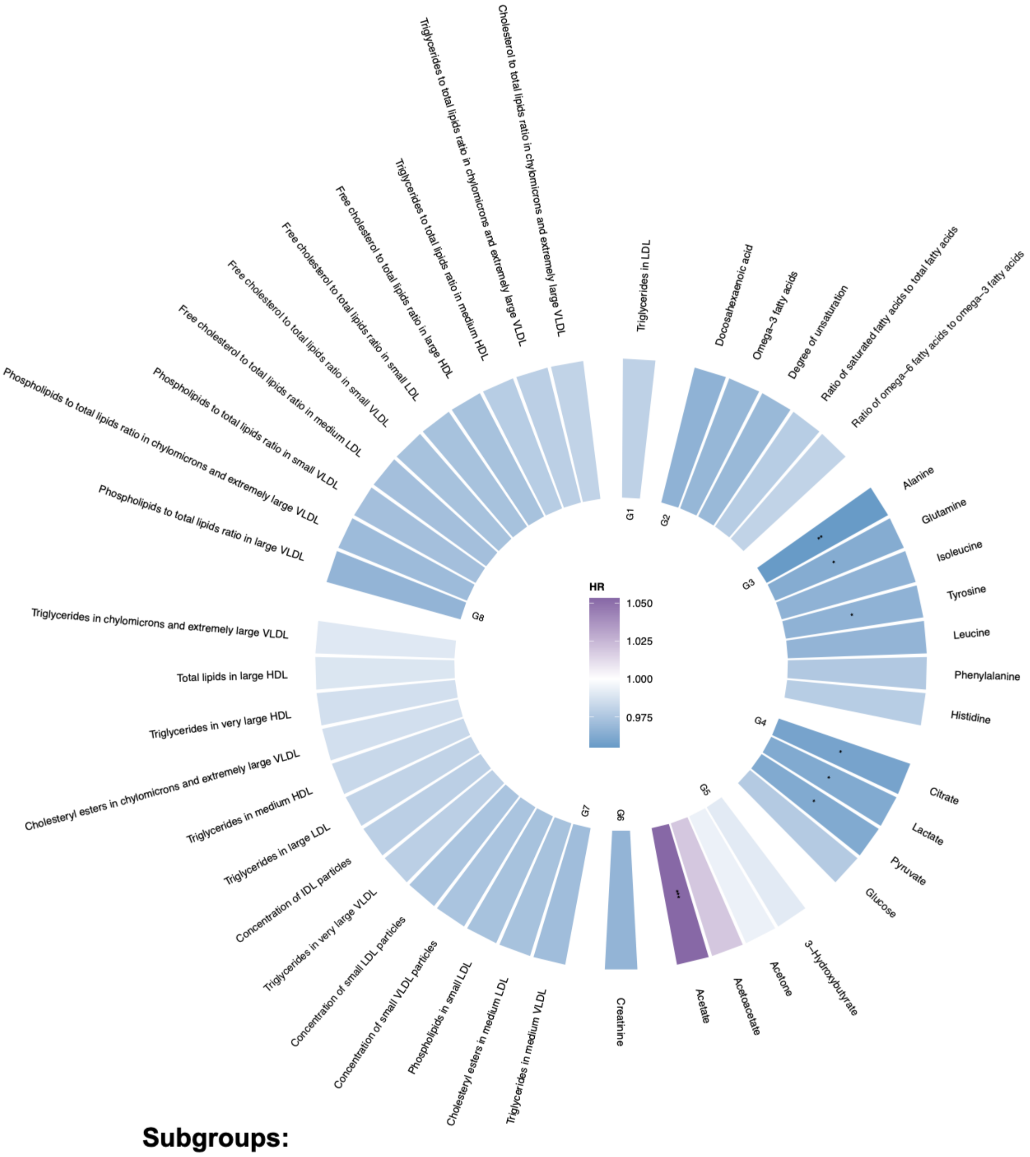

Subgroups:

G1 Triglycerides
G2 Fatty acids
G3 Amino acids
G4 Glycolysis related metabolites
G5 Ketone bodies
G6 Fluid balance
G7 Lipoprotein subclasses
G8 Relative lipoprotein lipid concentrations

Supplementary Fig. 6. The association between green space-related metabolites and depression among cancer survivors (N=21,507). The analysis was performed by Cox proportional hazards models with adjusting for age, sex and ethnicity, educational level, household income, employment status, body mass index, smoking status, drinking status, physical activity, diet and antidepressant use. The bars represent the estimated HRs of the metabolites on depression risk. *, raw P value <0.2; **, raw P value < 0.1; ***, raw P value < 0.05. All FDR-adjusted p-values exceeded 0.05. Exact P values are provided in the Source Data file, and all P values were two-sided. CI confidence interval, HR hazard ratio, HDL high-density lipoprotein, LDL low-density lipoprotein, VLDL very LDL. Source data are provided with this publication as a Source Data file.

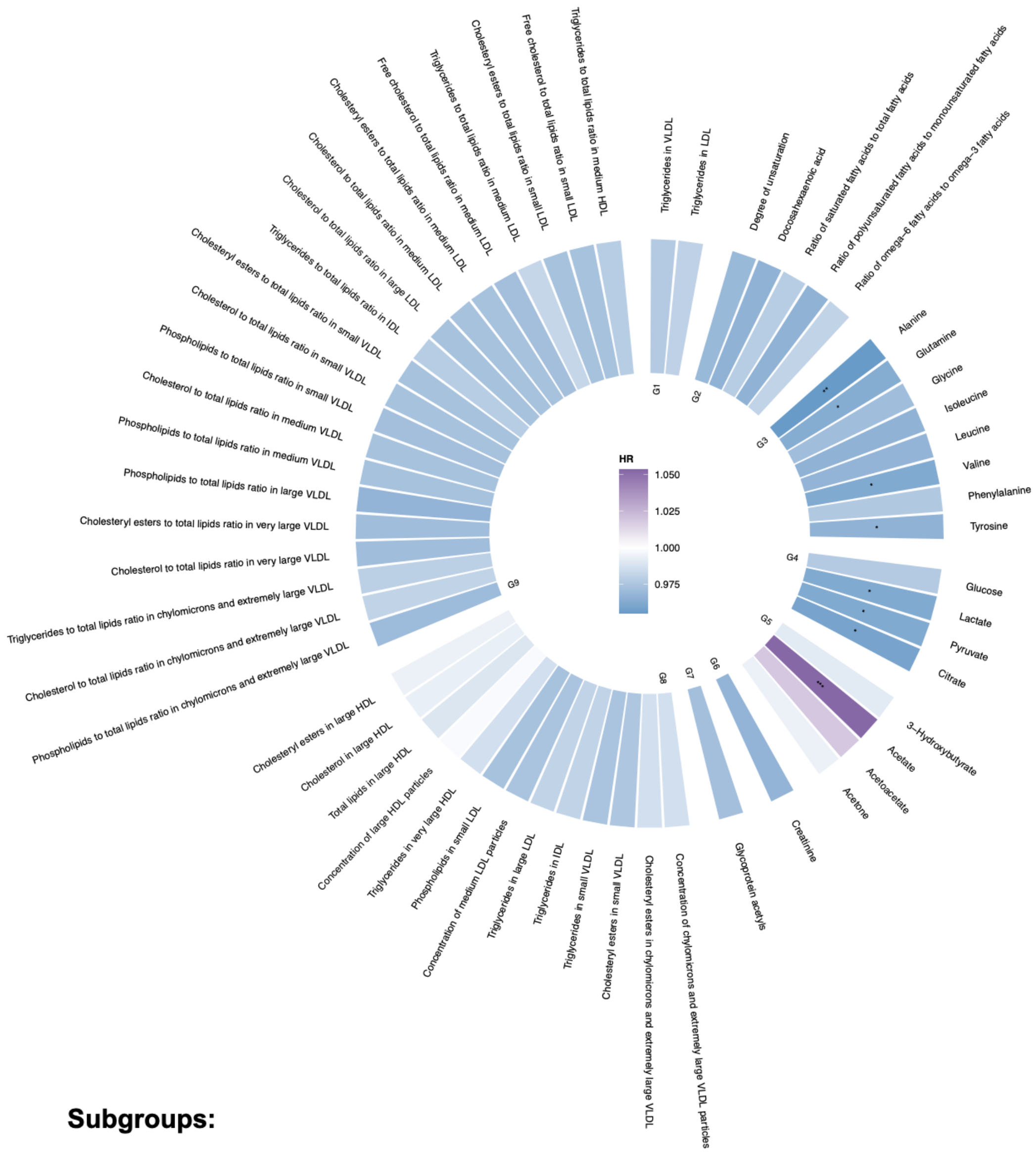

Subgroups:

G1 Triglycerides
G2 Fatty acids
G3 Amino acids
G4 Glycolysis related metabolites
G5 Ketone bodies
G6 Fluid balance
G7 Inflammation
G8 Lipoprotein subclasses
G9 Relative lipoprotein lipid concentrations

Supplementary Fig. 7. The association between natural environment-related metabolites and depression among cancer survivors (N=21,507). The analysis was performed by Cox proportional hazards models with adjusting for age, sex and ethnicity, educational level, household income, employment status, body mass index, smoking status, drinking status, physical activity, diet and antidepressant use. The bars represent the estimated HRs of the metabolites on depression risk. *, raw P value <0.2; **, raw P value < 0.1; ***, raw P value < 0.05. All FDR-adjusted p-values exceeded 0.05. Exact P values are provided in the Source Data file, and all P values were two-sided. CI confidence interval, HR hazard ratio, HDL high-density

lipoprotein, LDL low-density lipoprotein, VLDL very LDL. Source data are provided with this publication as a Source Data file.

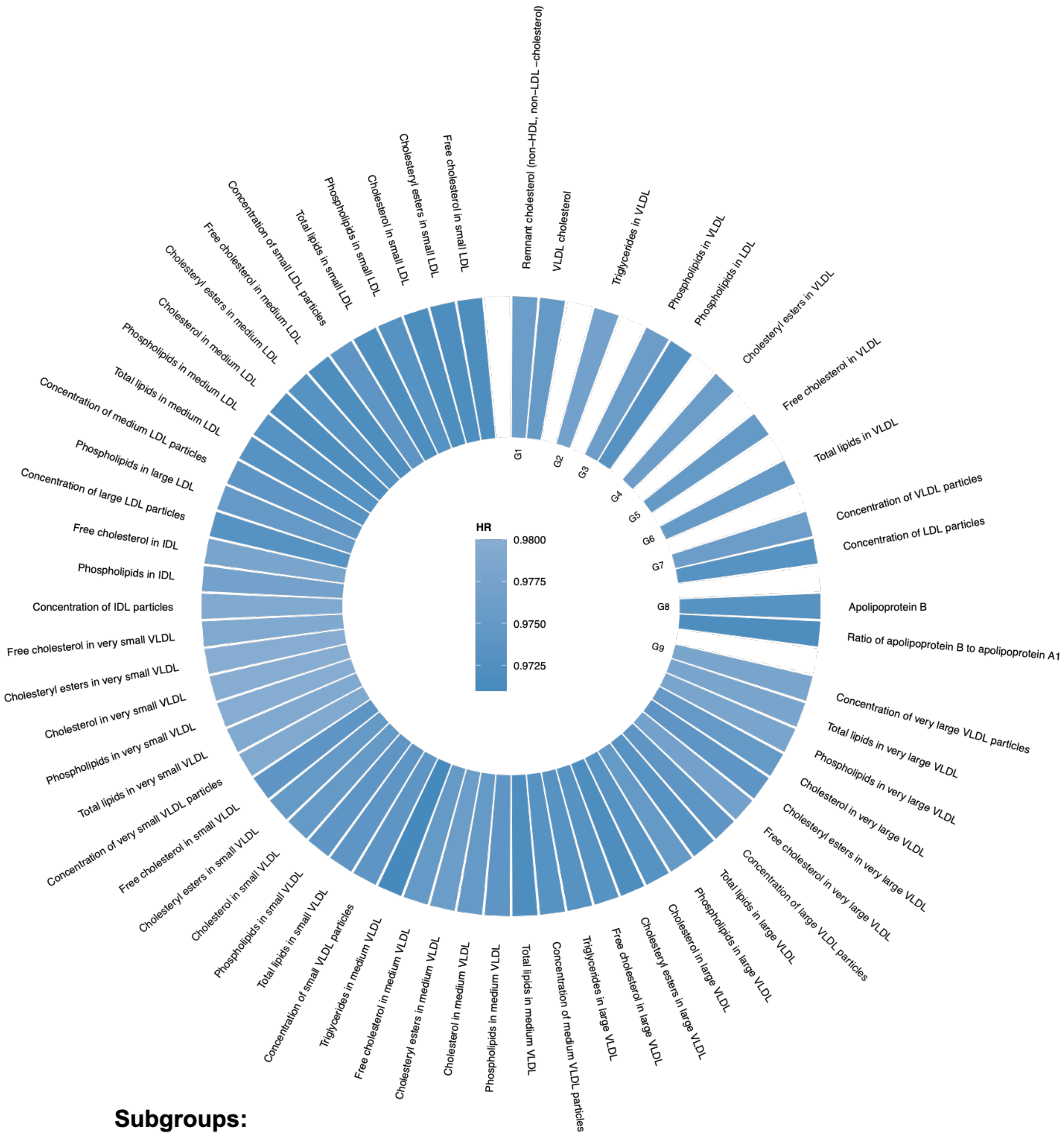

Subgroups:

G1 Cholesterol
G2 Triglycerides
G3 Phospholipids
G4 Cholesteryl esters
G5 Free cholesterol
G6 Total lipids
G7 Lipoprotein particle concentrations
G8 Apolipoproteins
G9 Lipoprotein subclasses

Supplementary Fig. 8. The association between air pollution score-related metabolites and depression among cancer survivors (N=21,507). The analysis was performed by Cox proportional hazards models with adjusting for age, sex and ethnicity, educational level, household income, employment status, body mass index, smoking status, drinking status, physical activity, diet and antidepressant use. The bars represent the estimated HRs of the metabolites on depression risk. *, raw P value <0.2; **, raw P value < 0.1; ***, raw P value < 0.05. All FDR-adjusted p-values exceeded 0.05. Exact P values are provided in the Source Data file, and all P values were two-sided. CI confidence interval, HR hazard ratio, HDL high-density

lipoprotein, LDL low-density lipoprotein, VLDL very LDL. Source data are provided with this publication as a Source Data file.

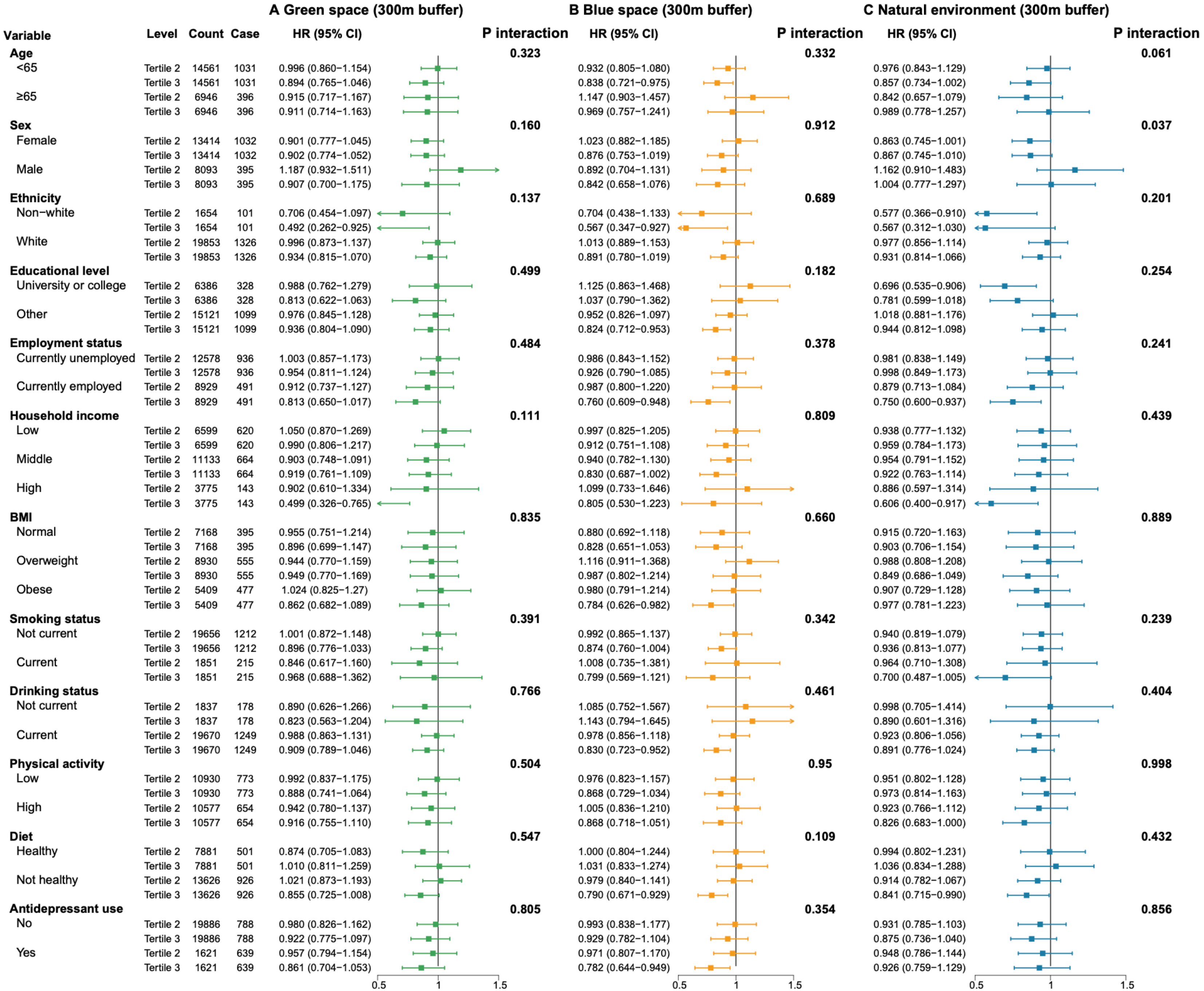

Variable	Level	Count	Case	A Green space (300m buffer) HR (95% CI)	P interaction	B Blue space (300m buffer) HR (95% CI)	P interaction	C Natural environment (300m buffer) HR (95% CI)	P interaction
**Age**					0.323		0.332		0.061
<65	Tertile 2	14561	1031	0.996 (0.860−1.154)		0.932 (0.805−1.080)		0.976 (0.843−1.129)	
	Tertile 3	14561	1031	0.894 (0.765−1.046)		0.838 (0.721−0.975)		0.857 (0.734−1.002)	
≥65	Tertile 2	6946	396	0.915 (0.717−1.167)		1.147 (0.903−1.457)		0.842 (0.657−1.079)	
	Tertile 3	6946	396	0.911 (0.714−1.163)		0.969 (0.757−1.241)		0.989 (0.778−1.257)	
**Sex**					0.160		0.912		0.037
Female	Tertile 2	13414	1032	0.901 (0.777−1.045)		1.023 (0.882−1.185)		0.863 (0.745−1.001)	
	Tertile 3	13414	1032	0.902 (0.774−1.052)		0.876 (0.753−1.019)		0.867 (0.745−1.010)	
Male	Tertile 2	8093	395	1.187 (0.932−1.511)		0.892 (0.704−1.131)		1.162 (0.910−1.483)	
	Tertile 3	8093	395	0.907 (0.700−1.175)		0.842 (0.658−1.076)		1.004 (0.777−1.297)	
**Ethnicity**					0.137		0.689		0.201
Non-white	Tertile 2	1654	101	0.706 (0.454−1.097)		0.704 (0.438−1.133)		0.577 (0.366−0.910)	
	Tertile 3	1654	101	0.492 (0.262−0.925)		0.567 (0.347−0.927)		0.567 (0.312−1.030)	
White	Tertile 2	19853	1326	0.996 (0.873−1.137)		1.013 (0.889−1.153)		0.977 (0.856−1.114)	
	Tertile 3	19853	1326	0.934 (0.815−1.070)		0.891 (0.780−1.019)		0.931 (0.814−1.066)	
**Educational level**					0.499		0.182		0.254
University or college	Tertile 2	6386	328	0.988 (0.762−1.279)		1.125 (0.863−1.468)		0.696 (0.535−0.906)	
	Tertile 3	6386	328	0.813 (0.622−1.063)		1.037 (0.790−1.362)		0.781 (0.599−1.018)	
Other	Tertile 2	15121	1099	0.976 (0.845−1.128)		0.952 (0.826−1.097)		1.018 (0.881−1.176)	
	Tertile 3	15121	1099	0.936 (0.804−1.090)		0.824 (0.712−0.953)		0.944 (0.812−1.098)	
**Employment status**					0.484		0.378		0.241
Currently unemployed	Tertile 2	12578	936	1.003 (0.857−1.173)		0.986 (0.843−1.152)		0.981 (0.838−1.149)	
	Tertile 3	12578	936	0.954 (0.811−1.124)		0.926 (0.790−1.085)		0.998 (0.849−1.173)	
Currently employed	Tertile 2	8929	491	0.912 (0.737−1.127)		0.987 (0.800−1.220)		0.879 (0.713−1.084)	
	Tertile 3	8929	491	0.813 (0.650−1.017)		0.760 (0.609−0.948)		0.750 (0.600−0.937)	
**Household income**					0.111		0.809		0.439
Low	Tertile 2	6599	620	1.050 (0.870−1.269)		0.997 (0.825−1.205)		0.938 (0.777−1.132)	
	Tertile 3	6599	620	0.990 (0.806−1.217)		0.912 (0.751−1.108)		0.959 (0.784−1.173)	
Middle	Tertile 2	11133	664	0.903 (0.748−1.091)		0.940 (0.782−1.130)		0.954 (0.791−1.152)	
	Tertile 3	11133	664	0.919 (0.761−1.109)		0.830 (0.687−1.002)		0.922 (0.763−1.114)	
High	Tertile 2	3775	143	0.902 (0.610−1.334)		1.099 (0.733−1.646)		0.886 (0.597−1.314)	
	Tertile 3	3775	143	0.499 (0.326−0.765)		0.805 (0.530−1.223)		0.606 (0.400−0.917)	
**BMI**					0.835		0.660		0.889
Normal	Tertile 2	7168	395	0.955 (0.751−1.214)		0.880 (0.692−1.118)		0.915 (0.720−1.163)	
	Tertile 3	7168	395	0.896 (0.699−1.147)		0.828 (0.651−1.053)		0.903 (0.706−1.154)	
Overweight	Tertile 2	8930	555	0.944 (0.770−1.159)		1.116 (0.911−1.368)		0.988 (0.808−1.208)	
	Tertile 3	8930	555	0.949 (0.770−1.169)		0.987 (0.802−1.214)		0.849 (0.686−1.049)	
Obese	Tertile 2	5409	477	1.024 (0.825−1.27)		0.980 (0.791−1.214)		0.907 (0.729−1.128)	
	Tertile 3	5409	477	0.862 (0.682−1.089)		0.784 (0.626−0.982)		0.977 (0.781−1.223)	
**Smoking status**					0.391		0.342		0.239
Not current	Tertile 2	19656	1212	1.001 (0.872−1.148)		0.992 (0.865−1.137)		0.940 (0.819−1.079)	
	Tertile 3	19656	1212	0.896 (0.776−1.033)		0.874 (0.760−1.004)		0.936 (0.813−1.077)	
Current	Tertile 2	1851	215	0.846 (0.617−1.160)		1.008 (0.735−1.381)		0.964 (0.710−1.308)	
	Tertile 3	1851	215	0.968 (0.688−1.362)		0.799 (0.569−1.121)		0.700 (0.487−1.005)	
**Drinking status**					0.766		0.461		0.404
Not current	Tertile 2	1837	178	0.890 (0.626−1.266)		1.085 (0.752−1.567)		0.998 (0.705−1.414)	
	Tertile 3	1837	178	0.823 (0.563−1.204)		1.143 (0.794−1.645)		0.890 (0.601−1.316)	
Current	Tertile 2	19670	1249	0.988 (0.863−1.131)		0.978 (0.856−1.118)		0.923 (0.806−1.056)	
	Tertile 3	19670	1249	0.909 (0.789−1.046)		0.830 (0.723−0.952)		0.891 (0.776−1.024)	
**Physical activity**					0.504		0.95		0.998
Low	Tertile 2	10930	773	0.992 (0.837−1.175)		0.976 (0.823−1.157)		0.951 (0.802−1.128)	
	Tertile 3	10930	773	0.888 (0.741−1.064)		0.868 (0.729−1.034)		0.973 (0.814−1.163)	
High	Tertile 2	10577	654	0.942 (0.780−1.137)		1.005 (0.836−1.210)		0.923 (0.766−1.112)	
	Tertile 3	10577	654	0.916 (0.755−1.110)		0.868 (0.718−1.051)		0.826 (0.683−1.000)	
**Diet**					0.547		0.109		0.432
Healthy	Tertile 2	7881	501	0.874 (0.705−1.083)		1.000 (0.804−1.244)		0.994 (0.802−1.231)	
	Tertile 3	7881	501	1.010 (0.811−1.259)		1.031 (0.833−1.274)		1.036 (0.834−1.288)	
Not healthy	Tertile 2	13626	926	1.021 (0.873−1.193)		0.979 (0.840−1.141)		0.914 (0.782−1.067)	
	Tertile 3	13626	926	0.855 (0.725−1.008)		0.790 (0.671−0.929)		0.841 (0.715−0.990)	
**Antidepressant use**					0.805		0.354		0.856
No	Tertile 2	19886	788	0.980 (0.826−1.162)		0.993 (0.838−1.177)		0.931 (0.785−1.103)	
	Tertile 3	19886	788	0.922 (0.775−1.097)		0.929 (0.782−1.104)		0.875 (0.736−1.040)	
Yes	Tertile 2	1621	639	0.957 (0.794−1.154)		0.971 (0.807−1.170)		0.948 (0.786−1.144)	
	Tertile 3	1621	639	0.861 (0.704−1.053)		0.782 (0.644−0.949)		0.926 (0.759−1.129)	

Supplementary Fig. 9. Associations between exposure to green space, blue space and natural environment at 300 m buffer and depression risk in stratified analyses.

HRs, 95% CIs, and P values were estimated using Cox proportional hazards regression based on Model 3, adjusted for age, sex and ethnicity, educational level, household income, employment status, body mass index, smoking status, drinking status, physical activity, diet and antidepressant use. Arrows indicate confidence intervals exceeding the plotting range. All P values were based on two-sided tests. CI confidence interval, HR hazard ratio. Source data are provided with this publication as a Source Data file.

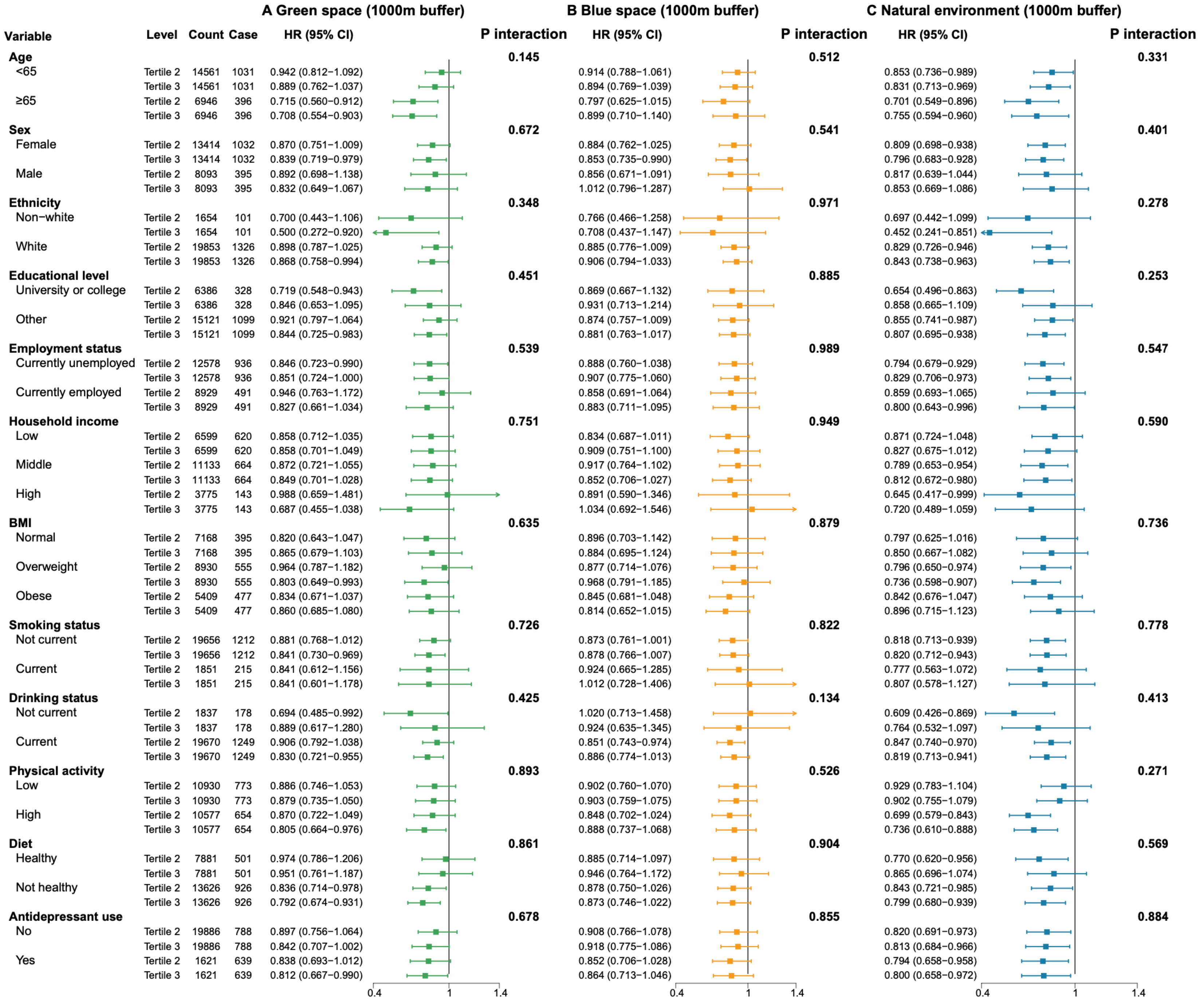

Variable	Level	Count	Case	A Green space HR (95% CI)	P interaction	B Blue space HR (95% CI)	P interaction	C Natural environment HR (95% CI)	P interaction
**Age**					**0.145**		**0.512**		**0.331**
<65	Tertile 2	14561	1031	0.942 (0.812−1.092)		0.914 (0.788−1.061)		0.853 (0.736−0.989)	
	Tertile 3	14561	1031	0.889 (0.762−1.037)		0.894 (0.769−1.039)		0.831 (0.713−0.969)	
≥65	Tertile 2	6946	396	0.715 (0.560−0.912)		0.797 (0.625−1.015)		0.701 (0.549−0.896)	
	Tertile 3	6946	396	0.708 (0.554−0.903)		0.899 (0.710−1.140)		0.755 (0.594−0.960)	
**Sex**					**0.672**		**0.541**		**0.401**
Female	Tertile 2	13414	1032	0.870 (0.751−1.009)		0.884 (0.762−1.025)		0.809 (0.698−0.938)	
	Tertile 3	13414	1032	0.839 (0.719−0.979)		0.853 (0.735−0.990)		0.796 (0.683−0.928)	
Male	Tertile 2	8093	395	0.892 (0.698−1.138)		0.856 (0.671−1.091)		0.817 (0.639−1.044)	
	Tertile 3	8093	395	0.832 (0.649−1.067)		1.012 (0.796−1.287)		0.853 (0.669−1.086)	
**Ethnicity**					**0.348**		**0.971**		**0.278**
Non-white	Tertile 2	1654	101	0.700 (0.443−1.106)		0.766 (0.466−1.258)		0.697 (0.442−1.099)	
	Tertile 3	1654	101	0.500 (0.272−0.920)		0.708 (0.437−1.147)		0.452 (0.241−0.851)	
White	Tertile 2	19853	1326	0.898 (0.787−1.025)		0.885 (0.776−1.009)		0.829 (0.726−0.946)	
	Tertile 3	19853	1326	0.868 (0.758−0.994)		0.906 (0.794−1.033)		0.843 (0.738−0.963)	
**Educational level**					**0.451**		**0.885**		**0.253**
University or college	Tertile 2	6386	328	0.719 (0.548−0.943)		0.869 (0.667−1.132)		0.654 (0.496−0.863)	
	Tertile 3	6386	328	0.846 (0.653−1.095)		0.931 (0.713−1.214)		0.858 (0.665−1.109)	
Other	Tertile 2	15121	1099	0.921 (0.797−1.064)		0.874 (0.757−1.009)		0.855 (0.741−0.987)	
	Tertile 3	15121	1099	0.844 (0.725−0.983)		0.881 (0.763−1.017)		0.807 (0.695−0.938)	
**Employment status**					**0.539**		**0.989**		**0.547**
Currently unemployed	Tertile 2	12578	936	0.846 (0.723−0.990)		0.888 (0.760−1.038)		0.794 (0.679−0.929)	
	Tertile 3	12578	936	0.851 (0.724−1.000)		0.907 (0.775−1.060)		0.829 (0.706−0.973)	
Currently employed	Tertile 2	8929	491	0.946 (0.763−1.172)		0.858 (0.691−1.064)		0.859 (0.693−1.065)	
	Tertile 3	8929	491	0.827 (0.661−1.034)		0.883 (0.711−1.095)		0.800 (0.643−0.996)	
**Household income**					**0.751**		**0.949**		**0.590**
Low	Tertile 2	6599	620	0.858 (0.712−1.035)		0.834 (0.687−1.011)		0.871 (0.724−1.048)	
	Tertile 3	6599	620	0.858 (0.701−1.049)		0.909 (0.751−1.100)		0.827 (0.675−1.012)	
Middle	Tertile 2	11133	664	0.872 (0.721−1.055)		0.917 (0.764−1.102)		0.789 (0.653−0.954)	
	Tertile 3	11133	664	0.849 (0.701−1.028)		0.852 (0.706−1.027)		0.812 (0.672−0.980)	
High	Tertile 2	3775	143	0.988 (0.659−1.481)		0.891 (0.590−1.346)		0.645 (0.417−0.999)	
	Tertile 3	3775	143	0.687 (0.455−1.038)		1.034 (0.692−1.546)		0.720 (0.489−1.059)	
**BMI**					**0.635**		**0.879**		**0.736**
Normal	Tertile 2	7168	395	0.820 (0.643−1.047)		0.896 (0.703−1.142)		0.797 (0.625−1.016)	
	Tertile 3	7168	395	0.865 (0.679−1.103)		0.884 (0.695−1.124)		0.850 (0.667−1.082)	
Overweight	Tertile 2	8930	555	0.964 (0.787−1.182)		0.877 (0.714−1.076)		0.796 (0.650−0.974)	
	Tertile 3	8930	555	0.803 (0.649−0.993)		0.968 (0.791−1.185)		0.736 (0.598−0.907)	
Obese	Tertile 2	5409	477	0.834 (0.671−1.037)		0.845 (0.681−1.048)		0.842 (0.676−1.047)	
	Tertile 3	5409	477	0.860 (0.685−1.080)		0.814 (0.652−1.015)		0.896 (0.715−1.123)	
**Smoking status**					**0.726**		**0.822**		**0.778**
Not current	Tertile 2	19656	1212	0.881 (0.768−1.012)		0.873 (0.761−1.001)		0.818 (0.713−0.939)	
	Tertile 3	19656	1212	0.841 (0.730−0.969)		0.878 (0.766−1.007)		0.820 (0.712−0.943)	
Current	Tertile 2	1851	215	0.841 (0.612−1.156)		0.924 (0.665−1.285)		0.777 (0.563−1.072)	
	Tertile 3	1851	215	0.841 (0.601−1.178)		1.012 (0.728−1.406)		0.807 (0.578−1.127)	
**Drinking status**					**0.425**		**0.134**		**0.413**
Not current	Tertile 2	1837	178	0.694 (0.485−0.992)		1.020 (0.713−1.458)		0.609 (0.426−0.869)	
	Tertile 3	1837	178	0.889 (0.617−1.280)		0.924 (0.635−1.345)		0.764 (0.532−1.097)	
Current	Tertile 2	19670	1249	0.906 (0.792−1.038)		0.851 (0.743−0.974)		0.847 (0.740−0.970)	
	Tertile 3	19670	1249	0.830 (0.721−0.955)		0.886 (0.774−1.013)		0.819 (0.713−0.941)	
**Physical activity**					**0.893**		**0.526**		**0.271**
Low	Tertile 2	10930	773	0.886 (0.746−1.053)		0.902 (0.760−1.070)		0.929 (0.783−1.104)	
	Tertile 3	10930	773	0.879 (0.735−1.050)		0.903 (0.759−1.075)		0.902 (0.755−1.079)	
High	Tertile 2	10577	654	0.870 (0.722−1.049)		0.848 (0.702−1.024)		0.699 (0.579−0.843)	
	Tertile 3	10577	654	0.805 (0.664−0.976)		0.888 (0.737−1.068)		0.736 (0.610−0.888)	
**Diet**					**0.861**		**0.904**		**0.569**
Healthy	Tertile 2	7881	501	0.974 (0.786−1.206)		0.885 (0.714−1.097)		0.770 (0.620−0.956)	
	Tertile 3	7881	501	0.951 (0.761−1.187)		0.946 (0.764−1.172)		0.865 (0.696−1.074)	
Not healthy	Tertile 2	13626	926	0.836 (0.714−0.978)		0.878 (0.750−1.026)		0.843 (0.721−0.985)	
	Tertile 3	13626	926	0.792 (0.674−0.931)		0.873 (0.746−1.022)		0.799 (0.680−0.939)	
**Antidepressant use**					**0.678**		**0.855**		**0.884**
No	Tertile 2	19886	788	0.897 (0.756−1.064)		0.908 (0.766−1.078)		0.820 (0.691−0.973)	
	Tertile 3	19886	788	0.842 (0.707−1.002)		0.918 (0.775−1.086)		0.813 (0.684−0.966)	
Yes	Tertile 2	1621	639	0.838 (0.693−1.012)		0.852 (0.706−1.028)		0.794 (0.658−0.958)	
	Tertile 3	1621	639	0.812 (0.667−0.990)		0.864 (0.713−1.046)		0.800 (0.658−0.972)	

Supplementary Fig. 10. Associations between exposure to green space, blue space and natural environment at 1000 m buffer and depression risk in stratified analyses.

HRs, 95% CIs, and P values were estimated using Cox proportional hazards regression based on Model 3, adjusted for age, sex and ethnicity, educational level, household income, employment status, body mass index, smoking status, drinking status, physical activity, diet and antidepressant use. Arrows indicate confidence intervals exceeding the plotting range. All P values were based on two-sided tests. CI confidence interval, HR hazard ratio. Source data are provided with this publication as a Source Data file.

Variable	Level	Count	Case	A NO_2 (μg/m³) HR (95% CI)	P interaction	B NO_X (μg/m³) HR (95% CI)	P interaction	C $PM_{2.5}$ (μg/m³) HR (95% CI)	P interaction	D Air pollution score HR (95% CI)	P interaction
**Age**					**0.161**		**0.245**		**0.267**		**0.542**
<65	Tertile 2	14561	1031	1.080 (0.924−1.263)		1.005 (0.857−1.179)		1.006 (0.859−1.178)		1.022 (0.872−1.198)	
	Tertile 3	14561	1031	1.131 (0.968−1.322)		1.136 (0.974−1.326)		1.108 (0.950−1.291)		1.135 (0.972−1.324)	
≥65	Tertile 2	6946	396	0.910 (0.711−1.165)		1.080 (0.848−1.376)		0.854 (0.668−1.093)		1.015 (0.794−1.296)	
	Tertile 3	6946	396	1.196 (0.942−1.519)		1.174 (0.918−1.502)		1.133 (0.892−1.438)		1.218 (0.955−1.553)	
**Sex**					**0.486**		**0.458**		**0.871**		**0.091**
Female	Tertile 2	13414	1032	0.998 (0.853−1.168)		0.976 (0.834−1.142)		0.945 (0.807−1.106)		0.936 (0.799−1.097)	
	Tertile 3	13414	1032	1.159 (0.995−1.351)		1.154 (0.991−1.344)		1.124 (0.966−1.307)		1.174 (1.009−1.367)	
Male	Tertile 2	8093	395	1.098 (0.860−1.403)		1.164 (0.906−1.494)		0.998 (0.779−1.280)		1.243 (0.972−1.590)	
	Tertile 3	8093	395	1.112 (0.866−1.427)		1.128 (0.877−1.451)		1.073 (0.840−1.369)		1.098 (0.852−1.416)	
**Ethnicity**					**0.604**		**0.467**		**0.318**		**0.375**
Non−white	Tertile 2	1654	101	1.459 (0.764−2.786)		0.758 (0.403−1.428)		0.803 (0.425−1.519)		0.909 (0.470−1.757)	
	Tertile 3	1654	101	1.647 (0.903−3.006)		1.518 (0.874−2.635)		1.829 (1.023−3.270)		1.726 (0.968−3.076)	
White	Tertile 2	19853	1326	1.009 (0.881−1.155)		1.038 (0.906−1.190)		0.967 (0.845−1.108)		1.030 (0.899−1.180)	
	Tertile 3	19853	1326	1.119 (0.979−1.281)		1.119 (0.978−1.281)		1.069 (0.936−1.221)		1.121 (0.980−1.282)	
**Educational level**					**0.674**		**0.411**		**0.886**		**0.678**
University or college	Tertile 2	6386	328	0.857 (0.645−1.139)		0.795 (0.597−1.060)		0.831 (0.628−1.101)		0.850 (0.639−1.129)	
	Tertile 3	6386	328	1.056 (0.814−1.368)		1.144 (0.884−1.480)		1.064 (0.820−1.381)		1.095 (0.844−1.420)	
Other	Tertile 2	15121	1099	1.073 (0.924−1.247)		1.094 (0.940−1.272)		0.995 (0.855−1.157)		1.072 (0.921−1.247)	
	Tertile 3	15121	1099	1.168 (1.004−1.359)		1.145 (0.984−1.332)		1.119 (0.965−1.298)		1.174 (1.010−1.364)	
**Employment status**					**0.887**		**0.660**		**0.285**		**0.780**
Currently unemployed	Tertile 2	12578	936	0.961 (0.818−1.130)		0.969 (0.824−1.139)		0.844 (0.717−0.994)		0.959 (0.815−1.128)	
	Tertile 3	12578	936	1.063 (0.904−1.250)		1.045 (0.889−1.229)		1.013 (0.865−1.186)		1.060 (0.902−1.245)	
Currently employed	Tertile 2	8929	491	1.166 (0.926−1.468)		1.145 (0.908−1.445)		1.220 (0.970−1.534)		1.144 (0.907−1.442)	
	Tertile 3	8929	491	1.292 (1.036−1.613)		1.339 (1.073−1.671)		1.317 (1.054−1.646)		1.339 (1.074−1.671)	
**Household income**					**0.985**		**0.849**		**0.633**		**0.846**
Low	Tertile 2	6599	620	1.038 (0.844−1.277)		1.067 (0.863−1.318)		0.850 (0.691−1.045)		1.012 (0.821−1.246)	
	Tertile 3	6599	620	1.053 (0.858−1.292)		1.069 (0.870−1.313)		0.944 (0.776−1.148)		1.006 (0.822−1.232)	
Middle	Tertile 2	11133	664	0.996 (0.825−1.204)		1.001 (0.828−1.210)		1.047 (0.864−1.268)		1.002 (0.828−1.214)	
	Tertile 3	11133	664	1.228 (1.017−1.482)		1.221 (1.012−1.472)		1.264 (1.047−1.526)		1.297 (1.075−1.565)	
High	Tertile 2	3775	143	1.019 (0.669−1.551)		0.866 (0.571−1.313)		0.964 (0.642−1.449)		1.022 (0.675−1.550)	
	Tertile 3	3775	143	1.206 (0.813−1.789)		1.183 (0.799−1.751)		1.203 (0.810−1.785)		1.224 (0.825−1.815)	
**BMI**					**0.332**		**0.921**		**0.337**		**0.906**
Normal	Tertile 2	7168	395	1.021 (0.795−1.311)		0.988 (0.766−1.272)		0.981 (0.760−1.265)		1.005 (0.780−1.296)	
	Tertile 3	7168	395	1.146 (0.895−1.469)		1.208 (0.945−1.546)		1.240 (0.972−1.581)		1.240 (0.968−1.588)	
Overweight	Tertile 2	8930	555	1.130 (0.917−1.392)		1.063 (0.860−1.315)		0.859 (0.694−1.063)		0.992 (0.802−1.226)	
	Tertile 3	8930	555	1.181 (0.955−1.459)		1.169 (0.948−1.441)		1.111 (0.906−1.362)		1.169 (0.951−1.436)	
Obese	Tertile 2	5409	477	0.888 (0.703−1.121)		0.988 (0.784−1.246)		1.039 (0.826−1.307)		1.034 (0.819−1.306)	
	Tertile 3	5409	477	1.083 (0.867−1.353)		1.052 (0.839−1.319)		1.008 (0.803−1.265)		1.065 (0.848−1.338)	
**Smoking status**					**0.716**		**0.297**		**0.941**		**0.717**
Not current	Tertile 2	19656	1212	0.989 (0.859−1.139)		0.981 (0.851−1.131)		0.944 (0.818−1.088)		0.985 (0.854−1.136)	
	Tertile 3	19656	1212	1.135 (0.987−1.306)		1.160 (1.009−1.333)		1.113 (0.969−1.278)		1.152 (1.002−1.325)	
Current	Tertile 2	1851	215	1.257 (0.856−1.846)		1.321 (0.905−1.929)		1.037 (0.713−1.507)		1.241 (0.845−1.824)	
	Tertile 3	1851	215	1.219 (0.844−1.760)		1.103 (0.763−1.595)		1.118 (0.782−1.598)		1.211 (0.840−1.746)	
**Drinking status**					**0.541**		**0.371**		**0.448**		**0.434**
Not current	Tertile 2	1837	178	0.921 (0.616−1.377)		0.787 (0.516−1.199)		0.887 (0.592−1.329)		0.843 (0.557−1.277)	
	Tertile 3	1837	178	1.260 (0.867−1.833)		1.278 (0.871−1.876)		1.274 (0.877−1.850)		1.314 (0.903−1.913)	
Current	Tertile 2	19670	1249	1.037 (0.902−1.193)		1.068 (0.928−1.228)		0.974 (0.846−1.121)		1.050 (0.912−1.208)	
	Tertile 3	19670	1249	1.139 (0.990−1.309)		1.127 (0.981−1.295)		1.097 (0.956−1.259)		1.140 (0.992−1.310)	
**Physical activity**					**0.684**		**0.446**		**0.992**		**0.407**
Low	Tertile 2	10930	773	1.002 (0.837−1.200)		1.015 (0.845−1.220)		0.929 (0.774−1.113)		0.969 (0.807−1.164)	
	Tertile 3	10930	773	1.082 (0.906−1.292)		1.151 (0.962−1.376)		1.052 (0.882−1.255)		1.128 (0.945−1.346)	
High	Tertile 2	10577	654	1.043 (0.858−1.267)		1.031 (0.850−1.250)		0.991 (0.815−1.205)		1.069 (0.880−1.298)	
	Tertile 3	10577	654	1.213 (0.999−1.472)		1.134 (0.936−1.373)		1.174 (0.973−1.418)		1.178 (0.972−1.428)	
**Diet**					**0.483**		**0.039**		**0.438**		**0.091**
Healthy	Tertile 2	7881	501	0.883 (0.706−1.106)		0.780 (0.622−0.978)		0.850 (0.678−1.064)		0.785 (0.625−0.987)	
	Tertile 3	7881	501	1.052 (0.847−1.307)		0.959 (0.774−1.188)		0.991 (0.799−1.229)		1.033 (0.834−1.279)	
Not healthy	Tertile 2	13626	926	1.104 (0.938−1.301)		1.191 (1.009−1.405)		1.025 (0.869−1.209)		1.170 (0.992−1.380)	
	Tertile 3	13626	926	1.185 (1.006−1.396)		1.256 (1.065−1.481)		1.165 (0.991−1.368)		1.217 (1.033−1.435)	
**Antidepressant use**					**0.963**		**0.100**		**0.600**		**0.485**
No	Tertile 2	19886	788	1.008 (0.844−1.203)		1.130 (0.947−1.348)		1.000 (0.838−1.193)		1.076 (0.901−1.284)	
	Tertile 3	19886	788	1.150 (0.966−1.370)		1.151 (0.964−1.373)		1.095 (0.921−1.302)		1.156 (0.970−1.377)	
Yes	Tertile 2	1621	639	1.044 (0.856−1.273)		0.902 (0.737−1.105)		0.904 (0.738−1.107)		0.952 (0.778−1.165)	
	Tertile 3	1621	639	1.161 (0.954−1.414)		1.166 (0.961−1.415)		1.137 (0.938−1.378)		1.176 (0.969−1.429)	

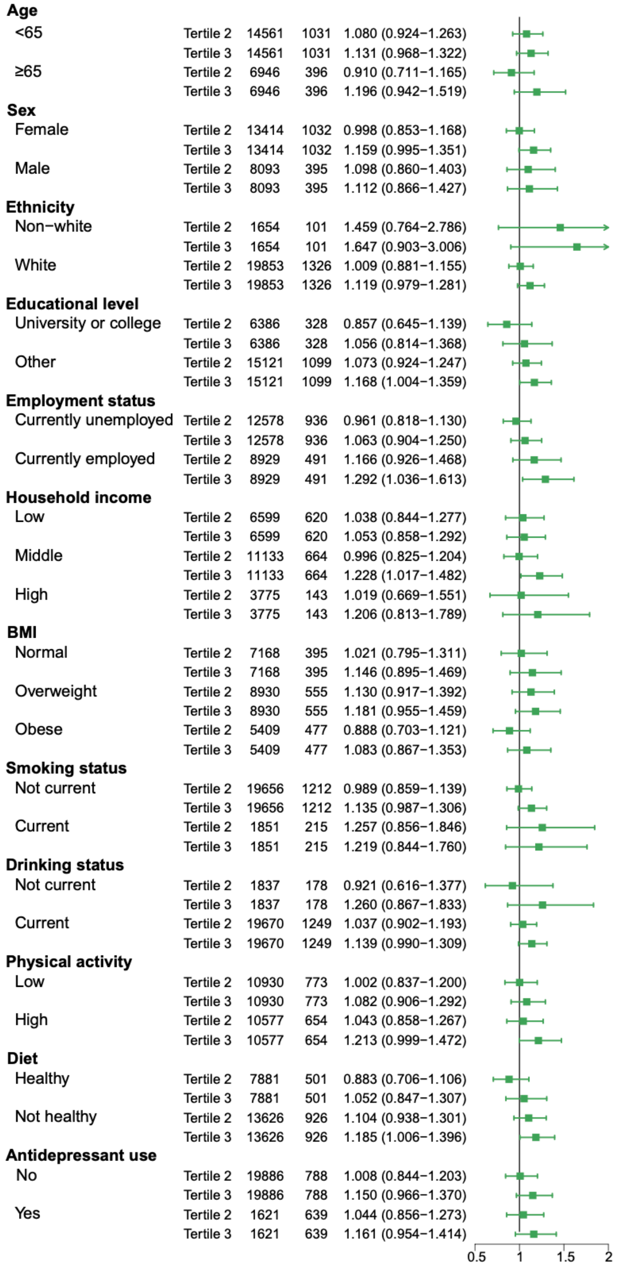

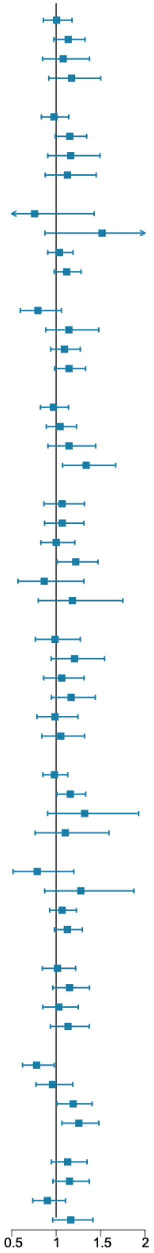

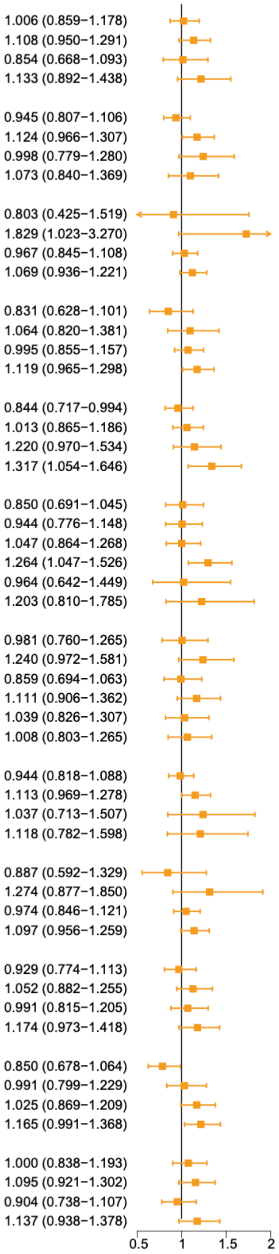

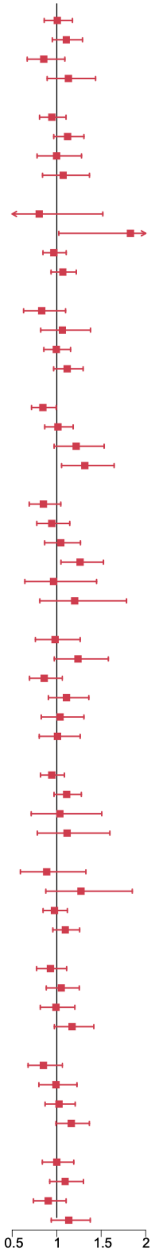

Supplementary Fig. 11. Associations between air pollutants and depression risk in stratified analyses.

HRs, 95% CIs, and P values were estimated using Cox proportional hazards regression based on Model 3, adjusted for age, sex and ethnicity, educational level, household income, employment status, body mass index, smoking status, drinking status, physical activity, diet and antidepressant use. Arrows indicate confidence intervals exceeding the plotting range. All P values were based on two-sided tests. CI confidence interval, HR hazard ratio, NO_2 nitrogen dioxide, NO_x nitrogen oxides, PM_{10} particulate matter (PM) with aerodynamic diameter ≤10 μm, $PM_{2.5}$ PM with aerodynamic diameter < 2.5 μm. Source data are provided with this publication as a Source Data file.

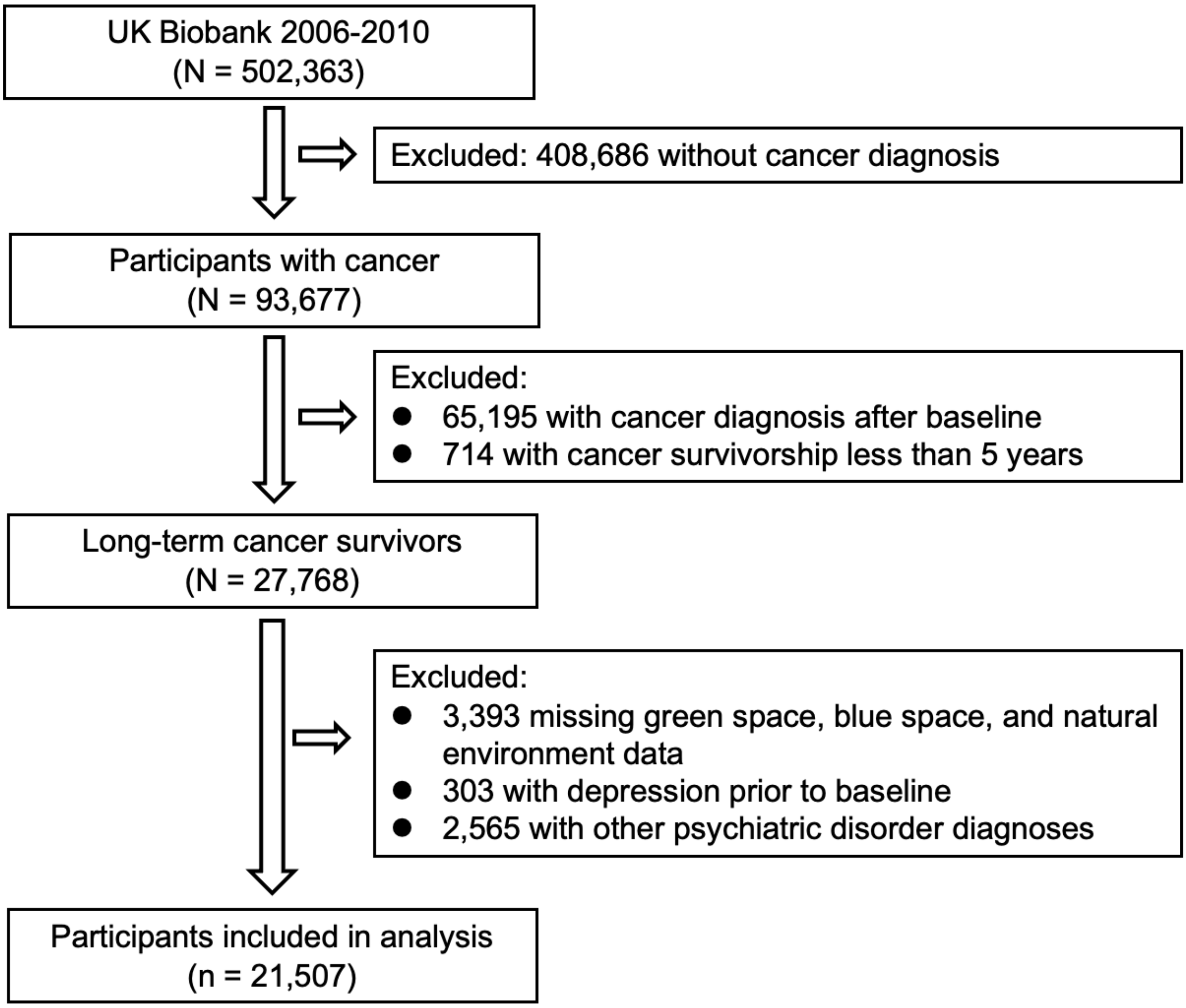

Supplementary Fig. 12. Flow chart of selection of study participants.

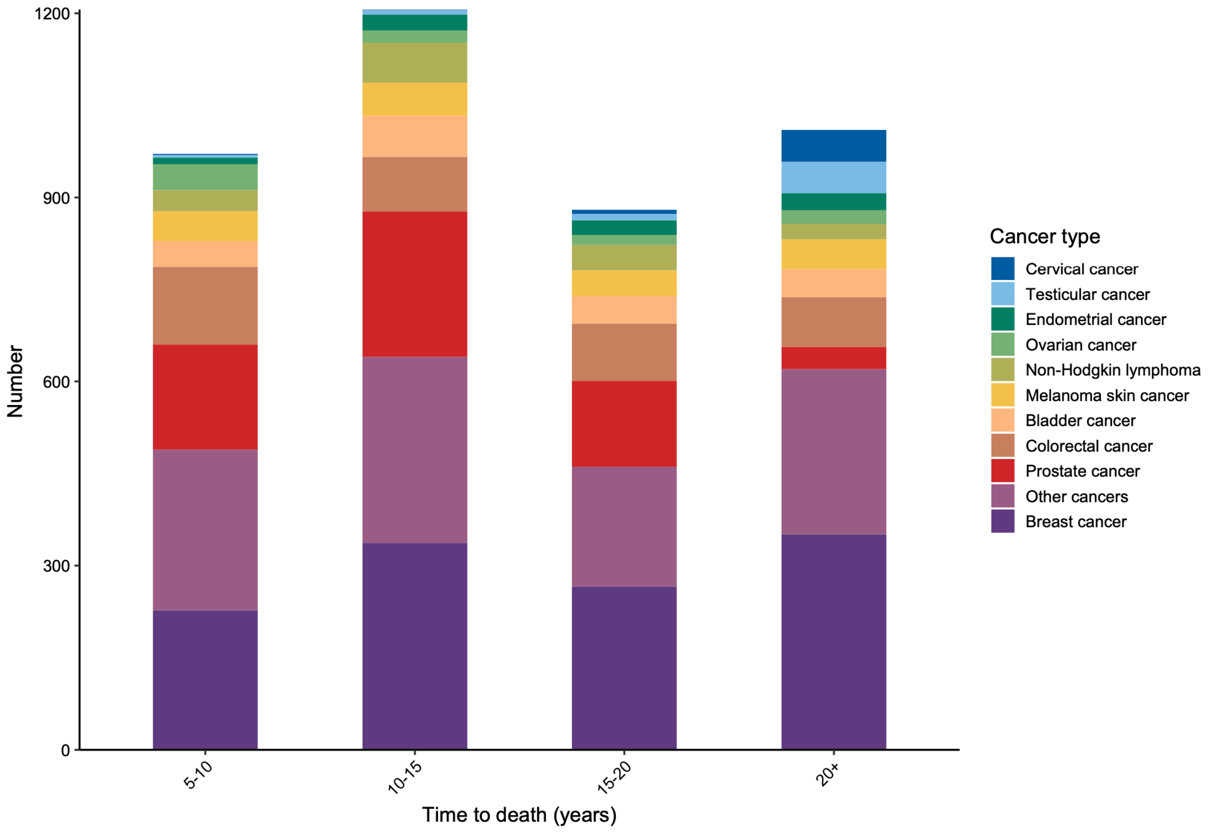

Supplementary Fig. 13. Distribution of cancer types and survival time among cancer survivors (N=21,507).

A total of 3,973 cases classified as "other cancer" included malignant neoplasms of lip, oral cavity and pharynx (467), kidney cancer (400), thyroid cancer (327), Hodgkin lymphoma (306), lymphoid leukemia (291), lung cancer (225), multiple myeloma (168), myeloid leukemia (157), malignant neoplasm of other connective and soft tissue (133), malignant neoplasm of larynx (132), oesophagus (118), brain (109), stomach (106), malignant neoplasm of eye and adnexa (96), secondary and unspecified malignant neoplasm of lymph nodes (72), peripheral and cutaneous T-cell lymphomas (58), malignant neoplasm of anus and anal canal (52), as well as other cancer types with fewer than 50 cases each. Source data are provided with this publication as a Source Data file.

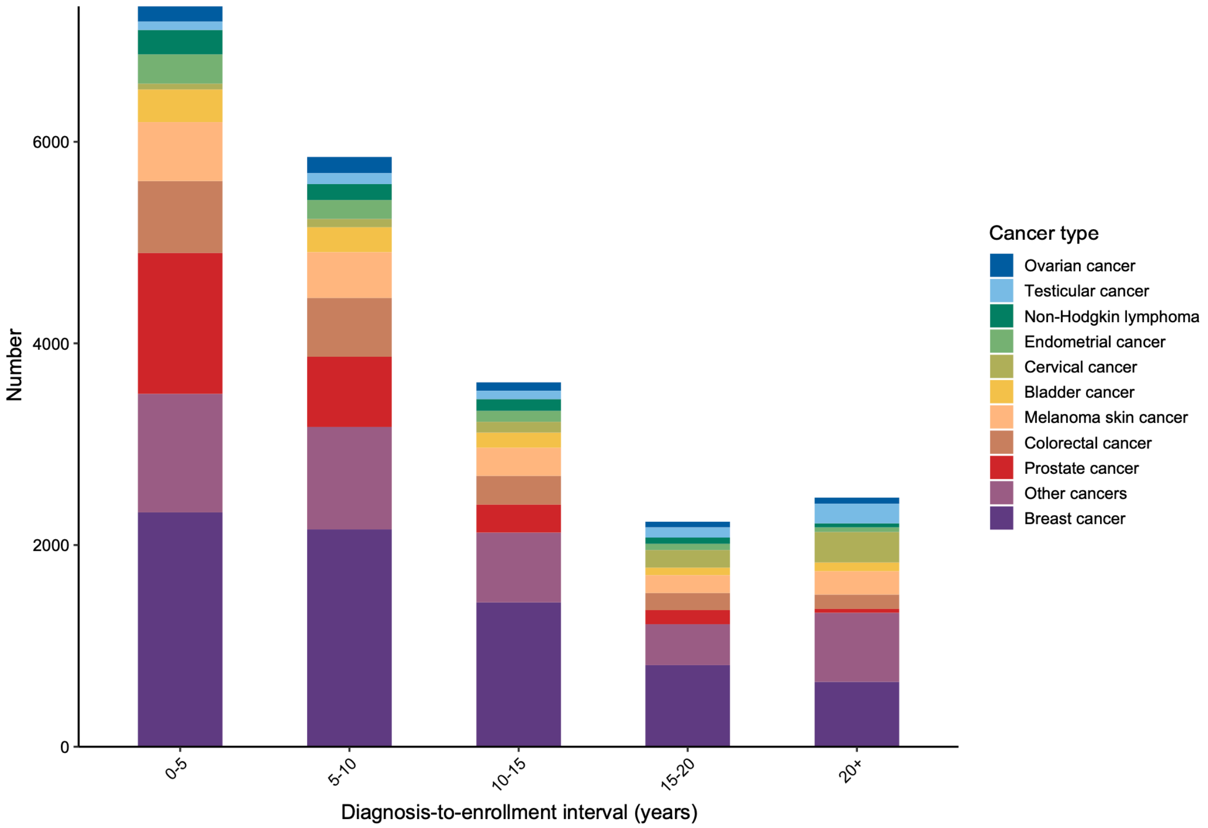

Supplementary Fig. 14. Distribution of cancer types and diagnosis-to-enrollment interval among cancer survivors (N=21,507).

A total of 3,973 cases classified as "other cancer" included malignant neoplasms of lip, oral cavity and pharynx (467), kidney cancer (400), thyroid cancer (327), Hodgkin lymphoma (306), lymphoid leukemia (291), lung cancer (225), multiple myeloma (168), myeloid leukemia (157), malignant neoplasm of other connective and soft tissue (133), malignant neoplasm of larynx (132), oesophagus (118), brain (109), stomach (106), malignant neoplasm of eye and adnexa (96), secondary and unspecified malignant neoplasm of lymph nodes (72), peripheral and cutaneous T-cell lymphomas (58), malignant neoplasm of anus and anal canal (52), as well as other cancer types with fewer than 50 cases each. Source data are provided with this publication as a Source Data file.

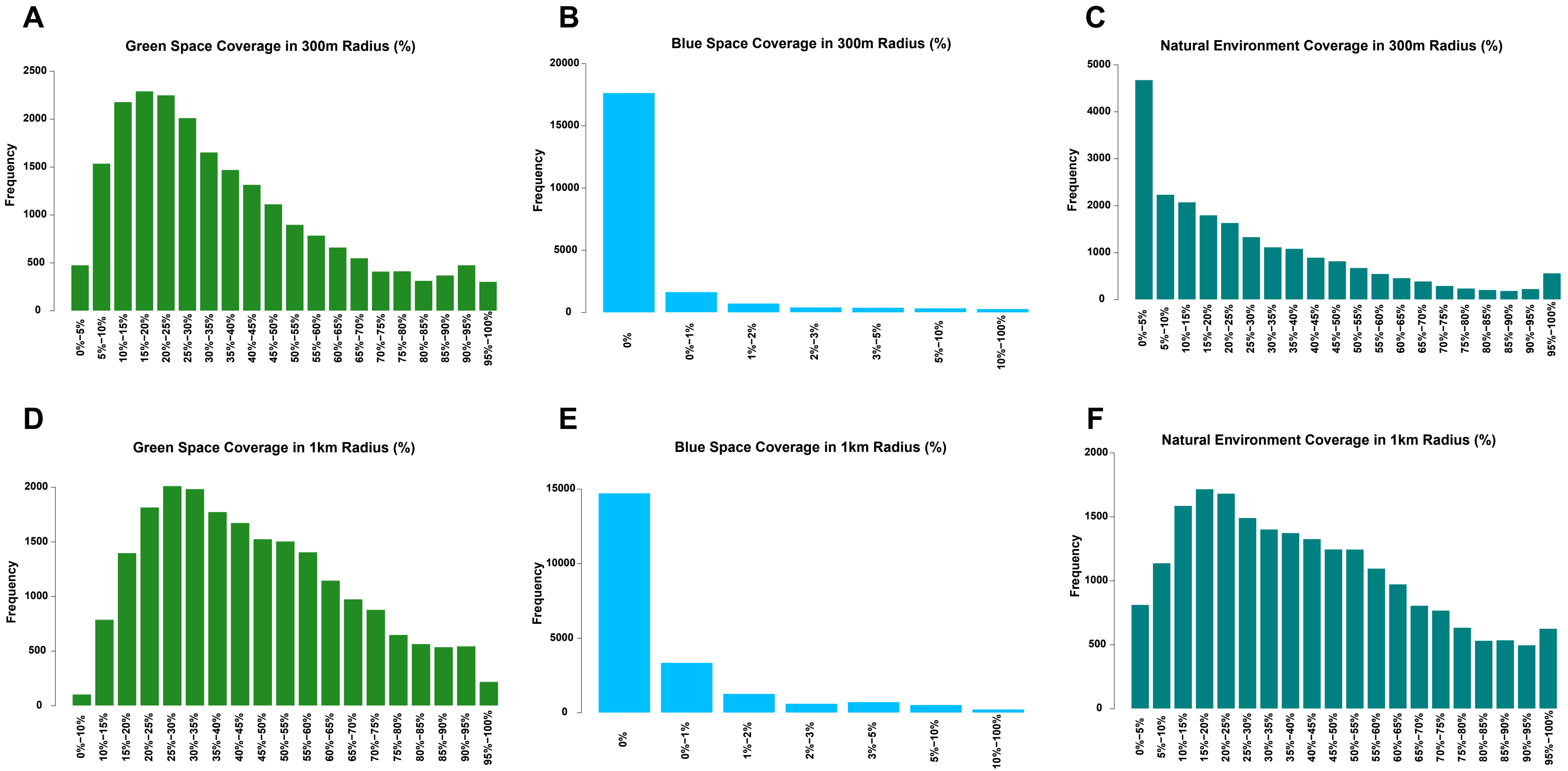

Supplementary Fig. 15. Distribution of cancer survivors across levels of green space, blue space, and natural environment exposure within 300 m (A, B and C) and 1000 m (D, E and F) buffers (N=21,507). Source data are provided with this publication as a Source Data file.

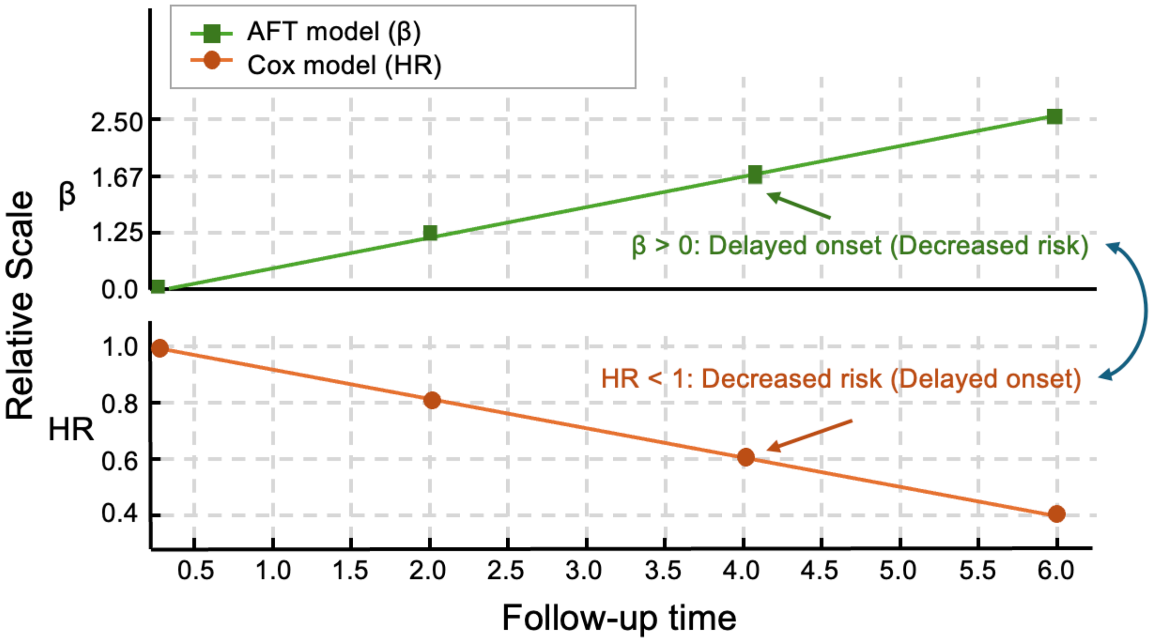

Supplementary Fig. 16. A brief schematic of β in the AFT model and HR in the Cox model interpretation.

The relationship between HR (from the Cox proportional hazards model) and β (from the AFT model) is generally complex and model-dependent. The schematic illustration of HR and β values provided here assumes an exponential distribution, under which HR and β can be approximately converted ($\beta \approx 1/HR$). AFT accelerated failure time, HR hazard ratio.

Supplementary Table 1. Distribution of residential green space, blue space and natural environment.

Exposures	Mean	Standard deviation	25th percentile	Median	75th percentile	Inter-quartile range
**Green space (%)**						
300 m buffer	35.79	23.36	17.59	30.01	49.09	31.51
1000 m buffer	45.71	21.60	28.12	42.63	60.63	32.51
**Blue space (%)**						
300 m buffer	0.92	3.14	0.01	0.14	0.62	0.61
1000 m buffer	1.27	2.54	0.20	0.52	1.29	1.09
**Natural environment (%)**						
300 m buffer	27.02	25.48	6.70	19.87	40.85	34.15
1000 m buffer	41.81	25.74	20.38	38.24	60.03	39.65

Supplementary Table 2. Correlation matrix of environmental exposures.

Exposures	Green space (300m buffer)	Green space (1000m buffer)	Blue space (300m buffer)	Blue space (1000m buffer)	Natural environment (300m buffer)	Natural environment (1000m buffer)	NO_2	NOx	PM_{10}	$PM_{2.5}$
Green space (300 m buffer)	1									
Green space (1000 m buffer)	0.853 ***	1								
Blue space (300 m buffer)	0.049 ***	0.020 **	1							
Blue space (1000 m buffer)	0.022 **	0.003	0.722 ***	1						
Natural environment (300 m buffer)	0.884 ***	0.761 ***	0.137 ***	0.086 ***	1					
Natural environment (1000 m buffer)	0.824 ***	0.968 ***	0.103 ***	0.112 ***	0.786 ***	1				
NO_2	-0.643 ***	-0.735 ***	-0.082 ***	-0.070 ***	-0.628 ***	-0.756 ***	1			
NO_x	-0.532 ***	-0.559 ***	-0.069 ***	-0.038 ***	-0.551 ***	-0.582 ***	0.922 ***	1		
PM_{10}	-0.419 ***	-0.390 ***	-0.052 ***	-0.013	-0.446 ***	-0.406 ***	0.520 ***	0.527 ***	1	
$PM_{2.5}$	-0.638 ***	-0.643 ***	-0.098 ***	-0.055 ***	-0.643 ***	-0.674 ***	0.864 ***	0.847 ***	0.548 ***	1
Depression	-0.016 *	-0.023 ***	-0.009	-0.007	-0.019 **	-0.030 ***	0.028 ***	0.030 ***	0.013	0.036 ***

Pearson correlation coefficients were used to assess linear associations between environmental exposures and air pollutants, and point-biserial correlation coefficients were used to assess the associations between environmental exposures, air pollutants, and depression. * P < 0.05; ** P < 0.01; *** P < 0.001. All P values were two-sided. NO_2 nitrogen dioxide, NO_x nitrogen oxides, PM_{10} particulate matter (PM) with aerodynamic diameter ≤10 μm, $PM_{2.5}$ PM with aerodynamic diameter < 2.5 μm.

Supplementary Table 3. Associations between green space, blue space, and natural environment at 300 m buffer and the risk of depression among cancer survivors (N=21,507).

**Exposures**	**Model 1**		**Model 2**		**Model 3**	
	**HR (95% CI)**	**P value**	**HR (95% CI)**	**P value**	**HR (95% CI)**	**P value**
**Green space**						
Tertile 1	Ref		Ref		Ref	
Tertile 2	1.077 (0.950-1.221)	0.244	1.024 (0.903-1.161)	0.714	0.971 (0.856-1.101)	0.646
Tertile 3	0.866 (0.760-0.988)	0.032	0.880 (0.772-1.004)	0.057	0.902 (0.791-1.029)	0.126
P trend		0.031		0.057		0.127
Per 5% increment	0.985 (0.974-0.996)	0.008	0.989 (0.977-1.000)	0.057	0.992 (0.98-1.004)	0.192
**Blue space**						
Tertile 1	Ref		Ref		Ref	
Tertile 2	0.956 (0.844-1.084)	0.485	0.970 (0.856-1.099)	0.629	0.987 (0.871-1.119)	0.840
Tertile 3	0.851 (0.749-0.968)	0.014	0.874 (0.769-0.994)	0.040	0.867 (0.763-0.986)	0.030
P trend		0.014		0.041		0.031
Per 5% increment	0.932 (0.842-1.031)	0.171	0.945 (0.856-1.043)	0.263	0.966 (0.869-1.073)	0.519
**Natural environment**						
Tertile 1	Ref		Ref		Ref	
Tertile 2	0.996 (0.879-1.129)	0.952	0.974 (0.859-1.104)	0.681	0.940 (0.829-1.066)	0.333
Tertile 3	0.842 (0.740-0.959)	0.009	0.871 (0.765-0.991)	0.037	0.903 (0.792-1.028)	0.124
P trend		0.009		0.037		0.123
Per 5% increment	0.984 (0.973-0.994)	0.003	0.989 (0.978-1.000)	0.042	0.992 (0.982-1.003)	0.176

HRs, 95% CIs, and P values were estimated using Cox proportional hazards regression models: Model 1 was adjusted for age, sex and ethnicity; Model 2 was further adjusted for educational level, household income and employment status; Model 3 was fully adjusted for body mass index, smoking status, drinking status, physical activity, diet and antidepressant use based on Model 2. All P values were two-sided. HR hazard ratio, CI confidence interval, Ref reference.

Supplementary Table 4. Associations between green space, blue space, and natural environment at 1000 m buffer and the risk of depression among cancer survivors (N=21,507).

**Exposures**	**Model 1**		**Model 2**		**Model 3**	
	**HR (95% CI)**	**P value**	**HR (95% CI)**	**P value**	**HR (95% CI)**	**P value**
**Green space**						
Tertile 1	Ref		Ref		Ref	
Tertile 2	0.933 (0.822-1.058)	0.278	0.901 (0.794-1.022)	0.106	0.878 (0.774-0.997)	0.044
Tertile 3	0.813 (0.714-0.925)	0.002	0.830 (0.729-0.945)	0.005	0.842 (0.739-0.960)	0.010
P trend		0.002		0.005		0.010
Per 5% increment	0.978 (0.966-0.989)	<0.001	0.980 (0.969-0.992)	0.001	0.983 (0.971-0.995)	0.006
**Blue space**						
Tertile 1	Ref		Ref		Ref	
Tertile 2	0.925 (0.815-1.049)	0.224	0.932 (0.822-1.058)	0.276	0.879 (0.774-0.997)	0.045
Tertile 3	0.892 (0.786-1.012)	0.077	0.902 (0.795-1.023)	0.109	0.896 (0.789-1.017)	0.088
P trend		0.076		0.108		0.085
Per 5% increment	0.970 (0.914-1.030)	0.322	0.976 (0.920-1.035)	0.414	0.995 (0.938-1.055)	0.868
**Natural environment**						
Tertile 1	Ref		Ref		Ref	
Tertile 2	0.812 (0.716-0.921)	0.001	0.796 (0.701-0.903)	<0.001	0.814 (0.717-0.923)	0.001
Tertile 3	0.739 (0.650-0.839)	<0.001	0.767 (0.675-0.873)	<0.001	0.818 (0.719-0.930)	0.002
P trend		<0.001		<0.001		0.002
Per 5% increment	0.975 (0.964-0.985)	<0.001	0.979 (0.968-0.989)	<0.001	0.983 (0.973-0.994)	0.002

HRs, 95% CIs, and P values were estimated using Cox proportional hazards regression models: Model 1 was adjusted for age, sex and ethnicity; Model 2 was further adjusted for educational level, household income and employment status; Model 3 was fully adjusted for body mass index, smoking status, drinking status, physical activity, diet and antidepressant use based on Model 2. All P values were two-sided. HR hazard ratio, CI confidence interval, Ref reference.

Supplementary Table 5. Mediation analysis of air pollutants and air pollution score in the association between green space, blue space, natural environment at 300 m buffer and depression risk (N=21,507).

Variable	Indirect effect		Direct effect		Proportion Mediated (%) *	
	β (95 % CI)	P value	β (95 % CI)	P value	β (95 % CI)	P value
**Green space**						
**NO_2**	1.97 (-0.28, 4.50)	0.088	0.12 (-4.04, 4.11)	0.942	94.48 (-672.86, 644.6)	0.270
**NO_x**	1.36 (-0.36, 3.04)	0.136	0.71 (-2.82, 4.13)	0.682	65.69 (-346.22, 716.91)	0.276
**$PM_{2.5}$**	1.60 (-0.44, 3.87)	0.138	0.42 (-3.60, 4.12)	0.816	79.32 (-620.31, 814.51)	0.300
**PM_{10}**	-0.45 (-1.70, 0.96)	0.472	2.46 (-0.88, 5.51)	0.158	-22.63 (-359.18, 194.34)	0.590
**Air pollution score**	1.87 (-0.43, 4.21)	0.120	0.19 (-3.42, 4.02)	0.920	90.79 (-723.76, 807.84)	0.258
**Blue space**						
**NO_2**	1.96 (-0.01, 3.95)	0.052	7.35 (-19.14, 50.47)	0.604	21.07 (-118.27, 219.19)	0.538
**NO_x**	1.49 (-0.08, 3.16)	0.074	7.90 (-18.28, 55.32)	0.550	15.90 (-121.18, 113.01)	0.538
**$PM_{2.5}$**	2.01 (-0.14, 4.61)	0.072	7.01 (-19.7, 51.85)	0.584	22.30 (-233.38, 177.95)	0.550
**PM_{10}**	-0.13 (-1.32, 1.05)	0.844	9.65 (-18.36, 49.9)	0.512	-1.39 (-48.56, 45.16)	0.874
**Air pollution score**	2.01 (0.06, 3.97)	0.048	7.21 (-19.44, 53.7)	0.582	21.79 (-129.44, 184.86)	0.520
**Natural environment**						
**NO_2**	1.74 (-0.32, 3.76)	0.112	0.26 (-3.30, 3.72)	0.840	86.81 (-452.57, 1014.78)	0.230
**NO_x**	1.30 (-0.37, 2.96)	0.140	0.69 (-2.68, 3.90)	0.722	65.31 (-576.94, 965.58)	0.286
**$PM_{2.5}$**	1.46 (-0.68, 3.52)	0.164	0.47 (-3.45, 3.75)	0.794	75.59 (-794.29, 907.54)	0.350
**PM_{10}**	-0.51 (-2.02, 0.67)	0.380	2.43 (-0.60, 5.90)	0.118	-26.54 (-388.45, 311.94)	0.548
**Air pollution score**	1.72 (-0.33, 3.71)	0.116	0.26 (-3.31, 3.74)	0.854	86.81 (-513.22, 832.75)	0.256

Mediation analysis was performed using an accelerated failure time model, with adjustment for age, sex and ethnicity, educational level, household income, employment status, body mass index, smoking status, drinking status, physical activity, diet and antidepressant use. The regression coefficient (β) represents the effect of exposure on log time to depression onset, where positive values indicate delayed onset (lower risk) and negative values indicate earlier onset (higher risk). * The proportion mediated was quantified as the ratio of the mediation effect to the total effect. All P values were two-sided. NO_2 nitrogen dioxide, NO_x nitrogen oxides, PM_{10} particulate matter (PM) with aerodynamic diameter

≤10 μm, $PM_{2.5}$ PM with aerodynamic diameter < 2.5 μm.

Supplementary Table 6. Mediation analysis of air pollutants and air pollution score in the association between green space, blue space, natural environment at 1000 m buffer and depression risk (N=21,507).

Variable	Indirect effect		Direct effect		Proportion Mediated (%) *	
	β (95 % CI)	P value	β (95 % CI)	P value	β (95 % CI)	P value
**Green space**						
**NO_2**	0.50 (-2.48, 3.67)	0.754	3.83 (-0.37, 7.77)	0.076	11.62 (-81.59, 113.25)	0.750
**NO_x**	0.69 (-1.26, 2.59)	0.472	3.64 (0.14, 6.80)	0.038	15.88 (-45.9, 87.97)	0.476
**$PM_{2.5}$**	0.39 (-1.87, 2.55)	0.772	3.93 (0.08, 7.32)	0.050	9.10 (-50.85, 101.74)	0.770
**PM_{10}**	-0.70 (-2.04, 0.48)	0.234	4.96 (1.78, 8.02)	0.004	-16.52 (-83.23, 13.96)	0.244
**Air pollution score**	0.65 (-2.23, 3.28)	0.608	3.67 (-0.55, 7.44)	0.080	15.10 (-76.95, 126.8)	0.618
**Blue space**						
**NO_2**	2.10 (0.26, 4.30)	0.018	0.71 (-26.23, 38.24)	0.918	74.68 (-168.22, 232.93)	0.802
**NO_x**	1.05 (-0.03, 2.30)	0.060	1.97 (-24.64, 41.53)	0.850	34.69 (-128.31, 87.81)	0.834
**$PM_{2.5}$**	1.43 (0.01, 2.97)	0.048	1.19 (-24.16, 35.40)	0.906	54.60 (-168.01, 172.44)	0.832
**PM_{10}**	-0.04 (-0.51, 0.43)	0.840	2.43 (-22.34, 36.76)	0.804	-1.64 (-15.39, 12.83)	0.990
**Air pollution score**	1.68 (0.03, 3.29)	0.048	1.18 (-25.39, 37.43)	0.892	58.7 (-143.03, 186.24)	0.780
**Natural environment**						
**NO_2**	0.02 (-2.96, 2.78)	0.998	3.99 (0.41, 8.06)	0.034	0.56 (-95.80, 85.60)	0.998
**NO_x**	0.45 (-1.36, 2.30)	0.606	3.56 (0.44, 6.53)	0.028	11.29 (-40.95, 72.55)	0.604
**$PM_{2.5}$**	0.05 (-2.23, 2.18)	0.986	3.96 (0.67, 7.21)	0.016	1.30 (-77.05, 65.11)	0.986
**PM_{10}**	-0.72 (-1.84, 0.34)	0.208	4.66 (2.15, 7.25)	<0.001	-18.15 (-71.08, 8.88)	0.212
**Air pollution score**	0.27 (-2.14, 2.65)	0.832	3.75 (0.22, 7.06)	0.042	6.67 (-73.06, 91.50)	0.830

Mediation analysis was performed using an accelerated failure time model, with adjustment for age, sex and ethnicity, educational level, household income, employment status, body mass index, smoking status, drinking status, physical activity, diet and antidepressant use. The regression coefficient (β) represents the effect of exposure on log time to depression onset, where positive values indicate delayed onset (lower risk) and negative values indicate earlier onset (higher risk). * The proportion mediated was quantified as the ratio of the mediation effect to the total effect. All P values were two-sided. NO_2 nitrogen dioxide, NO_x nitrogen oxides, PM_{10} particulate matter (PM) with aerodynamic diameter

≤10 μm, $PM_{2.5}$ PM with aerodynamic diameter < 2.5 μm.

Supplementary Table 7. Associations between green space-related metabolic signature and green space and air pollutants.

Variable	Estimated changes (95% CIs) of green space-related metabolic signature				
	Per 5% increment	Tertile 1	Tertile 2	Tertile 3	P for trend
**Green space (1000 m buffers)**	0.018 (0.015, 0.021)	Ref	0.088 (0.055, 0.121)	0.192 (0.159, 0.225)	<0.001
**NO_2**	-0.009 (-0.010, -0.007)	Ref	-0.045 (-0.077, -0.012)	-0.157 (-0.19, -0.124)	<0.001
**NO_x**	-0.003 (-0.004, -0.002)	Ref	-0.047 (-0.079, -0.014)	-0.11 (-0.143, -0.077)	<0.001
**$PM_{2.5}$**	-0.029 (-0.036, -0.022)	Ref	-0.051 (-0.084, -0.019)	-0.093 (-0.125, -0.06)	<0.001
**PM_{10}**	-0.04 (-0.053, -0.027)	Ref	-0.071 (-0.103, -0.038)	-0.134 (-0.167, -0.102)	<0.001
**Air pollution score**	-0.032 (-0.04, -0.024)	Ref	-0.047 (-0.08, -0.015)	-0.119 (-0.152, -0.086)	<0.001

Multivariable linear regression models were used to estimate associations, with adjustment for age, sex, ethnicity, educational level, household income, employment status, body mass index, smoking status, drinking status, physical activity, diet and antidepressant use. All P values were two-sided. CI confidence interval, Ref reference, NO_2 nitrogen dioxide, NO_x nitrogen oxides, PM particulate matter, $PM_{2.5}$ PM with aerodynamic diameter ≤2.5 μm, PM10 PM with aerodynamic diameter ≤10 μm.

Supplementary Table 8. Associations between nature environment-related metabolic signature and nature environment and air pollutants.

**Variable**	**Estimated changes (95% CIs) of nature environment-related metabolic signature**				
	Per 5% increment	Tertile 1	Tertile 2	Tertile 3	P for trend
**Natural environment (1000 m buffers)**	0.018 (0.015, 0.021)	Ref	0.13 (0.097, 0.163)	0.234 (0.201, 0.267)	<0.001
**NO_2**	-0.01 (-0.012, -0.009)	Ref	-0.054 (-0.087, -0.022)	-0.195 (-0.228, -0.162)	<0.001
**NO_x**	-0.004 (-0.004, -0.003)	Ref	-0.069 (-0.101, -0.036)	-0.147 (-0.18, -0.113)	<0.001
**$PM_{2.5}$**	-0.03 (-0.038, -0.023)	Ref	-0.07 (-0.103, -0.037)	-0.131 (-0.164, -0.098)	<0.001
**PM_{10}**	-0.051 (-0.064, -0.038)	Ref	-0.086 (-0.118, -0.053)	-0.149 (-0.182, -0.116)	<0.001
**Air pollution score**	-0.04 (-0.048, -0.032)	Ref	-0.062 (-0.094, -0.029)	-0.156 (-0.189, -0.123)	<0.001

Multivariable linear regression models were used to estimate associations, with adjustment for age, sex, ethnicity, educational level, household income, employment status, body mass index, smoking status, drinking status, physical activity, diet and antidepressant use. All P values were two-sided. CI confidence interval, Ref reference, NO_2 nitrogen dioxide, NO_x nitrogen oxides, PM particulate matter, $PM_{2.5}$ PM with aerodynamic diameter ≤2.5 μm, PM10 PM with aerodynamic diameter ≤10 μm.

Supplementary Table 9. Associations between air pollution score-related metabolic signature and green space, nature environment, and air pollutants.

Variable	Estimated changes (95% CIs) of APS-related metabolic signature				
	Per 5% increment	Tertile 1	Tertile 2	Tertile 3	P for trend
**Green space (1000 m buffers)**	-0.012 (-0.015, -0.009)	Ref	-0.061 (-0.094, -0.029)	-0.124 (-0.156, -0.091)	<0.001
**Natural environment**	-0.011 (-0.013, -0.008)	Ref	-0.106 (-0.139, -0.073)	-0.145 (-0.178, -0.112)	<0.001
**NO_2**	0.008 (0.006, 0.009)	Ref	0.036 (0.004, 0.068)	0.135 (0.102, 0.168)	<0.001
**NO_x**	0.003 (0.002, 0.004)	Ref	0.041 (0.009, 0.073)	0.106 (0.073, 0.138)	<0.001
**$PM_{2.5}$**	0.042 (0.029, 0.055)	Ref	0.032 (0, 0.065)	0.097 (0.065, 0.13)	<0.001
**PM_{10}**	0.016 (0.009, 0.023)	Ref	0.042 (0.009, 0.074)	0.076 (0.044, 0.109)	<0.001
**Air pollution score**	0.031 (0.023, 0.039)	Ref	0.032 (-0.001, 0.064)	0.114 (0.081, 0.146)	<0.001

Multivariable linear regression models were used to estimate associations, with adjustment for age, sex, ethnicity, educational level, household income, employment status, body mass index, smoking status, drinking status, physical activity, diet and antidepressant use. All P values were two-sided. CI confidence interval, Ref reference, NO_2 nitrogen dioxide, NO_x nitrogen oxides, PM particulate matter, $PM_{2.5}$ PM with aerodynamic diameter ≤2.5 μm, PM_{10} PM with aerodynamic diameter ≤10 μm.

Supplementary Table 10. Associations between environmental exposures at 300 m buffer and depression risk in breast cancer patients (N=7,365).

**Exposures**	**Model 1**		**Model 2**		**Model 3**	
	**HR (95% CI)**	**P**	**HR (95% CI)**	**P**	**HR (95% CI)**	**P**
**Green space**						
Tertile 1	Ref		Ref		Ref	
Tertile 2	0.962 (0.782-1.183)	0.711	0.901 (0.732-1.110)	0.328	0.857 (0.696-1.055)	0.146
Tertile 3	0.907 (0.738-1.116)	0.357	0.899 (0.730-1.106)	0.313	0.876 (0.712-1.079)	0.214
P trend		0.356		0.315		0.217
Per 5% increment	0.986 (0.968-1.004)	0.128	0.988 (0.970-1.007)	0.202	0.987 (0.968-1.006)	0.182
**Blue space**						
Tertile 1	Ref		Ref		Ref	
Tertile 2	0.839 (0.684-1.030)	0.093	0.849 (0.692-1.042)	0.117	0.869 (0.708-1.067)	0.180
Tertile 3	0.827 (0.673-1.017)	0.071	0.839 (0.682-1.031)	0.095	0.802 (0.652-0.986)	0.036
P trend		0.068		0.091		0.035
Per 5% increment	0.970 (0.844-1.115)	0.666	0.974 (0.851-1.114)	0.700	0.998 (0.860-1.158)	0.978
**Natural environment**						
Tertile 1	Ref		Ref		Ref	
Tertile 2	0.934 (0.759-1.150)	0.520	0.910 (0.739-1.120)	0.372	0.877 (0.712-1.080)	0.216
Tertile 3	0.913 (0.743-1.122)	0.387	0.929 (0.755-1.143)	0.487	0.920 (0.747-1.133)	0.433
P trend		0.387		0.486		0.431
Per 5% increment	0.985 (0.969-1.003)	0.096	0.988 (0.971-1.006)	0.192	0.989 (0.972-1.007)	0.237

HRs, 95% CIs, and P values were estimated using Cox proportional hazards regression models: Model 1 was adjusted for age, sex and ethnicity; Model 2 was further adjusted for educational level, household income and employment status; Model 3 was fully adjusted for body mass index,

smoking status, drinking status, physical activity, diet and antidepressant use based on Model 2. All P values were two-sided. CI confidence interval, HR hazard ratio, Ref reference.

Supplementary Table 11. Associations between environmental exposures at 1000 m buffer and depression risk in breast cancer patients (N=7,365).

**Exposures**	**Model 1**		**Model 2**		**Model 3**	
	**HR (95% CI)**	**P**	**HR (95% CI)**	**P**	**HR (95% CI)**	**P**
**Green space**						
Tertile 1	Ref		Ref		Ref	
Tertile 2	0.939 (0.765-1.152)	0.545	0.887 (0.722-1.090)	0.255	0.875 (0.712-1.075)	0.205
Tertile 3	0.806 (0.653-0.995)	0.045	0.801 (0.649-0.990)	0.040	0.781 (0.631-0.966)	0.023
P trend		0.044		0.040		0.022
Per 5% increment	0.976 (0.958-0.994)	0.009	0.976 (0.957-0.995)	0.012	0.974 (0.955-0.993)	0.008
**Blue space**						
Tertile 1	Ref		Ref		Ref	
Tertile 2	0.865 (0.703-1.065)	0.172	0.870 (0.707-1.071)	0.190	0.807 (0.656-0.994)	0.044
Tertile 3	0.903 (0.737-1.106)	0.325	0.901 (0.735-1.104)	0.314	0.882 (0.720-1.081)	0.227
P trend		0.317		0.307		0.219
Per 5% increment	0.995 (0.903-1.096)	0.914	1.000 (0.910-1.099)	0.998	1.014 (0.924-1.113)	0.772
**Natural environment**						
Tertile 1	Ref		Ref		Ref	
Tertile 2	0.746 (0.607-0.918)	0.006	0.724 (0.588-0.891)	0.002	0.743 (0.603-0.914)	0.005
Tertile 3	0.733 (0.597-0.900)	0.003	0.743 (0.604-0.913)	0.005	0.761 (0.618-0.936)	0.010
P trend		0.003		0.005		0.009
Per 5% increment	0.972 (0.956-0.989)	0.001	0.974 (0.957-0.991)	0.003	0.975 (0.958-0.992)	0.005

HRs, 95% CIs, and P values were estimated using Cox proportional hazards regression models: Model 1 was adjusted for age, sex and ethnicity; Model 2 was further adjusted for educational level, household income and employment status; Model 3 was fully adjusted for body mass index,

smoking status, drinking status, physical activity, diet and antidepressant use based on Model 2. All P values were two-sided. CI confidence interval, HR hazard ratio, Ref reference.

Supplementary Table 12. Associations between environmental exposures at 300 m buffer and depression risk in melanoma skin cancer patients (N=1,721).

Exposures	Model 1		Model 2		Model 3	
	HR (95% CI)	P	HR (95% CI)	P	HR (95% CI)	P
**Green space**						
Tertile 1	Ref		Ref		Ref	
Tertile 2	0.837 (0.664-1.056)	0.133	0.818 (0.648-1.032)	0.090	0.865 (0.679-1.101)	0.238
Tertile 3	0.732 (0.580-0.926)	0.009	0.750 (0.593-0.949)	0.016	0.813 (0.638-1.035)	0.092
P trend		0.009		0.017		0.094
Per 5% increment	0.980 (0.960-1.000)	0.052	0.983 (0.963-1.004)	0.116	0.989 (0.968-1.010)	0.298
**Blue space**						
Tertile 1	Ref		Ref		Ref	
Tertile 2	0.935 (0.742-1.179)	0.571	0.952 (0.755-1.200)	0.677	0.954 (0.750-1.213)	0.699
Tertile 3	0.882 (0.697-1.117)	0.297	0.911 (0.719-1.154)	0.439	1.029 (0.807-1.312)	0.816
P trend		0.297		0.439		0.818
Per 5% increment	0.928 (0.776-1.109)	0.410	0.943 (0.792-1.122)	0.506	0.987 (0.835-1.166)	0.875
**Natural environment**						
Tertile 1	Ref		Ref		Ref	
Tertile 2	0.971 (0.767-1.230)	0.809	0.962 (0.759-1.219)	0.748	0.904 (0.707-1.156)	0.421
Tertile 3	0.834 (0.658-1.058)	0.135	0.859 (0.677-1.089)	0.210	0.873 (0.684-1.114)	0.273
P trend		0.126		0.203		0.281
Per 5% increment	0.980 (0.961-0.999)	0.038	0.983 (0.964-1.003)	0.097	0.986 (0.967-1.006)	0.184

HRs, 95% CIs, and P values were estimated using Cox proportional hazards regression models: Model 1 was adjusted for age, sex and ethnicity; Model 2 was further adjusted for educational level, household income and employment status; Model 3 was fully adjusted for body mass index,

smoking status, drinking status, physical activity, diet, antidepressant use and sun exposure based on Model 2. All P values were two-sided. CI confidence interval, HR hazard ratio, Ref reference.

Supplementary Table 13. Associations between environmental exposures at 1000 m buffer and depression risk in melanoma skin cancer patients (N=1,721).

Exposures	Model 1		Model 2		Model 3	
	HR (95% CI)	P	HR (95% CI)	P	HR (95% CI)	P
**Green space**						
Tertile 1	Ref		Ref		Ref	
Tertile 2	0.879 (0.697-1.109)	0.278	0.873 (0.691-1.103)	0.254	0.908 (0.712-1.158)	0.436
Tertile 3	0.724 (0.570-0.919)	0.008	0.745 (0.586-0.947)	0.016	0.799 (0.623-1.024)	0.076
P trend		0.008		0.016		0.074
Per 5% increment	0.973 (0.953-0.994)	0.012	0.977 (0.956-0.998)	0.029	0.981 (0.960-1.003)	0.091
**Blue space**						
Tertile 1	Ref		Ref		Ref	
Tertile 2	0.948 (0.753-1.193)	0.647	0.957 (0.760-1.205)	0.709	0.952 (0.750-1.208)	0.684
Tertile 3	0.924 (0.730-1.169)	0.511	0.944 (0.746-1.195)	0.633	0.991 (0.778-1.261)	0.939
P trend		0.509		0.630		0.932
Per 5% increment	0.971 (0.879-1.072)	0.561	0.977 (0.885-1.078)	0.644	1.000 (0.905-1.104)	0.993
**Natural environment**						
Tertile 1	Ref		Ref		Ref	
Tertile 2	0.814 (0.644-1.030)	0.086	0.818 (0.647-1.035)	0.094	0.837 (0.656-1.068)	0.153
Tertile 3	0.706 (0.557-0.895)	0.004	0.734 (0.578-0.932)	0.011	0.780 (0.610-0.998)	0.048
P trend		0.004		0.012		0.052
Per 5% increment	0.973 (0.954-0.992)	0.005	0.976 (0.957-0.996)	0.017	0.981 (0.962-1.001)	0.068

HRs, 95% CIs, and P values were estimated using Cox proportional hazards regression models: Model 1 was adjusted for age, sex and ethnicity; Model 2 was further adjusted for educational level, household income and employment status; Model 3 was fully adjusted for body mass index,

smoking status, drinking status, physical activity, diet, antidepressant use and sun exposure based on Model 2. All P values were two-sided. CI confidence interval, HR hazard ratio, Ref reference.

Supplementary Table 14. Associations between environmental exposures at 300 m buffer and depression risk in non-melanoma skin cancer patients (N=14,896).

Exposures	Model 1		Model 2		Model 3	
	HR (95% CI)	P	HR (95% CI)	P	HR (95% CI)	P
**Green space**						
Tertile 1	Ref		Ref		Ref	
Tertile 2	1.166 (0.710-1.916)	0.544	1.154 (0.701-1.898)	0.574	1.247 (0.738-2.107)	0.409
Tertile 3	0.772 (0.457-1.303)	0.332	0.774 (0.459-1.306)	0.338	0.867 (0.503-1.494)	0.608
P trend		0.326		0.331		0.608
Per 5% increment	0.991 (0.949-1.034)	0.668	0.992 (0.950-1.036)	0.726	1.003 (0.960-1.047)	0.907
**Blue space**						
Tertile 1	Ref		Ref		Ref	
Tertile 2	1.026 (0.639-1.648)	0.916	0.990 (0.615-1.591)	0.966	1.325 (0.799-2.197)	0.276
Tertile 3	0.575 (0.332-0.998)	0.049	0.570 (0.329-0.988)	0.045	0.648 (0.361-1.162)	0.145
P trend		0.054		0.049		0.171
Per 5% increment	0.965 (0.719-1.295)	0.811	0.972 (0.722-1.309)	0.852	0.961 (0.657-1.407)	0.839
**Natural environment**						
Tertile 1	Ref		Ref		Ref	
Tertile 2	1.209 (0.729-2.006)	0.462	1.177 (0.709-1.953)	0.530	1.318 (0.771-2.255)	0.313
Tertile 3	0.856 (0.505-1.450)	0.562	0.862 (0.509-1.461)	0.582	0.969 (0.560-1.675)	0.909
P trend		0.529		0.552		0.867
Per 5% increment	1.005 (0.965-1.046)	0.817	1.007 (0.967-1.049)	0.729	1.009 (0.968-1.052)	0.670

HRs, 95% CIs, and P values were estimated using Cox proportional hazards regression models: Model 1 was adjusted for age, sex and ethnicity; Model 2 was further adjusted for educational level, household income and employment status; Model 3 was fully adjusted for body mass index, smoking status, drinking status, physical activity, diet, antidepressant use and sun exposure based on Model 2. Non-melanoma skin cancer

patients were not included in the main cancer survivor cohort, but were analyzed separately in sensitivity analyses. All P values were two-sided.
CI confidence interval, HR hazard ratio, Ref reference.

Supplementary Table 15. Associations between environmental exposures at 1000 m buffer and depression risk in non-melanoma skin cancer patients (N=14,896).

Exposures	Model 1		Model 2		Model 3	
	HR (95% CI)	P	HR (95% CI)	P	HR (95% CI)	P
**Green space**						
Tertile 1	Ref		Ref		Ref	
Tertile 2	0.871 (0.527-1.439)	0.590	0.842 (0.509-1.392)	0.502	0.999 (0.589-1.692)	0.996
Tertile 3	0.686 (0.409-1.150)	0.153	0.684 (0.408-1.147)	0.150	0.814 (0.471-1.408)	0.462
P trend		0.151		0.149		0.458
Per 5% increment	0.985 (0.943-1.029)	0.486	0.987 (0.944-1.031)	0.551	0.998 (0.953-1.044)	0.918
**Blue space**						
Tertile 1	Ref		Ref		Ref	
Tertile 2	0.828 (0.512-1.338)	0.440	0.825 (0.510-1.335)	0.434	0.603 (0.360-1.010)	0.054
Tertile 3	0.615 (0.365-1.038)	0.069	0.610 (0.361-1.030)	0.065	0.549 (0.319-0.946)	0.031
P trend		0.069		0.064		0.026
Per 5% increment	0.819 (0.582-1.152)	0.251	0.802 (0.567-1.136)	0.214	0.777 (0.512-1.179)	0.235
**Natural environment**						
Tertile 1	Ref		Ref		Ref	
Tertile 2	0.724 (0.433-1.211)	0.219	0.699 (0.417-1.172)	0.175	0.850 (0.492-1.467)	0.559
Tertile 3	0.706 (0.425-1.173)	0.179	0.715 (0.430-1.189)	0.196	0.803 (0.472-1.367)	0.419
P trend		0.194		0.217		0.427
Per 5% increment	0.986 (0.946-1.027)	0.489	0.988 (0.948-1.030)	0.568	0.997 (0.955-1.040)	0.880

HRs, 95% CIs, and P values were estimated using Cox proportional hazards regression models: Model 1 was adjusted for age, sex and ethnicity; Model 2 was further adjusted for educational level, household income and employment status; Model 3 was fully adjusted for body mass index, smoking status, drinking status, physical activity, diet, antidepressant use and sun exposure based on Model 2. Non-melanoma skin cancer

patients were not included in the main cancer survivor cohort, but were analyzed separately in sensitivity analyses. All P values were two-sided.
CI confidence interval, HR hazard ratio, Ref reference.

Supplementary Table 16. Associations between environmental exposures at 300 m buffer and depression risk in lung cancer patients (N=265).

Exposures	Model 1		Model 2		Model 3		Model 4	
	HR (95% CI)	P	HR (95% CI)	P	HR (95% CI)	P	HR (95% CI)	P
**Green space**								
Tertile 1	Ref		Ref		Ref		Ref	
Tertile 2	1.408 (0.444-4.468)	0.561	1.406 (0.443-4.464)	0.563	3.299 (0.784-13.877)	0.103	2.769 (0.554-13.833)	0.215
Tertile 3	1.391 (0.441-4.387)	0.573	1.423 (0.441-4.592)	0.555	3.441 (0.794-14.919)	0.099	5.089 (0.818-31.671)	0.081
P trend		0.563		0.543		0.099		0.080
Per 5% increment	1.030 (0.938-1.130)	0.541	1.031 (0.936-1.134)	0.538	1.072 (0.962-1.195)	0.210	1.140 (0.968-1.342)	0.117
**Blue space**								
Tertile 1	Ref		Ref		Ref		Ref	
Tertile 2	2.751 (0.738-10.26)	0.132	2.737 (0.720-10.397)	0.139	7.444 (1.492-37.140)	0.014	7.269 (1.124-47.008)	0.037
Tertile 3	2.140 (0.530-8.631)	0.285	2.226 (0.541-9.162)	0.267	2.545 (0.546-11.853)	0.234	1.596 (0.261-9.741)	0.613
P trend		0.321		0.290		0.311		0.748
Per 5% increment	0.889 (0.578-1.366)	0.590	0.896 (0.590-1.360)	0.606	0.841 (0.574-1.230)	0.372	0.756 (0.400-1.432)	0.391
**Natural environment**								
Tertile 1	Ref		Ref		Ref		Ref	
Tertile 2	0.763 (0.241-2.418)	0.646	0.674 (0.208-2.186)	0.511	0.763 (0.205-2.846)	0.687	0.875 (0.195-3.932)	0.862
Tertile 3	0.955 (0.315-2.898)	0.936	0.904 (0.294-2.779)	0.861	0.692 (0.195-2.460)	0.570	0.521 (0.107-2.537)	0.419
P trend		0.922		0.849		0.569		0.412
Per 5% increment	0.992 (0.896-1.099)	0.880	0.989 (0.890-1.098)	0.833	0.968 (0.855-1.097)	0.612	0.973 (0.829-1.142)	0.740

HRs, 95% CIs, and P values were estimated using Cox proportional hazards regression models: Model 1 was adjusted for age, sex and ethnicity; Model 2 was further adjusted for educational level, household income and employment status; Model 3 was additionally adjusted for body mass

index, smoking status, drinking status, physical activity, diet and antidepressant use based on Model 2; Model 4 was fully adjusted for all air pollutants based on Model 3. All P values were two-sided. CI confidence interval, HR hazard ratio, Ref reference.

Supplementary Table 17. Associations between environmental exposures at 1000 m buffer and depression risk in lung cancer patients (N=265).

Exposures	Model 1		Model 2		Model 3		Model 4	
	HR (95% CI)	P	HR (95% CI)	P	HR (95% CI)	P	HR (95% CI)	P
**Green space**								
Tertile 1	Ref		Ref		Ref		Ref	
Tertile 2	0.969 (0.318-2.953)	0.956	0.916 (0.301-2.791)	0.877	1.576 (0.436-5.700)	0.488	1.700 (0.321-8.995)	0.532
Tertile 3	0.905 (0.267-3.072)	0.873	0.868 (0.252-2.987)	0.822	1.284 (0.289-5.704)	0.742	0.987 (0.108-9.026)	0.990
P trend		0.874		0.821		0.716		0.986
Per 5% increment	0.985 (0.889-1.093)	0.782	0.983 (0.884-1.093)	0.751	1.015 (0.898-1.146)	0.815	1.027 (0.858-1.229)	0.770
**Blue space**								
Tertile 1	Ref		Ref		Ref		Ref	
Tertile 2	0.692 (0.244-1.964)	0.489	0.705 (0.246-2.018)	0.515	0.921 (0.290-2.923)	0.890	0.455 (0.115-1.795)	0.261
Tertile 3	0.357 (0.096-1.323)	0.123	0.356 (0.095-1.326)	0.124	0.205 (0.045-0.930)	0.040	0.098 (0.015-0.652)	0.016
P trend		0.115		0.117		0.047		0.015
Per 5% increment	0.717 (0.386-1.332)	0.292	0.715 (0.377-1.357)	0.305	0.505 (0.239-1.066)	0.073	0.424 (0.163-1.106)	0.079
**Natural environment**								
Tertile 1	Ref		Ref		Ref		Ref	
Tertile 2	1.388 (0.475-4.060)	0.549	1.331 (0.455-3.898)	0.602	1.916 (0.566-6.486)	0.296	1.982 (0.380-10.322)	0.417
Tertile 3	0.761 (0.210-2.755)	0.677	0.741 (0.199-2.754)	0.654	0.980 (0.211-4.551)	0.979	1.137 (0.141-9.180)	0.904
P trend		0.726		0.714		0.933		0.954
Per 5% increment	0.973 (0.882-1.074)	0.592	0.971 (0.879-1.074)	0.572	0.984 (0.876-1.104)	0.782	0.970 (0.813-1.157)	0.732

HRs, 95% CIs, and P values were estimated using Cox proportional hazards regression models: Model 1 was adjusted for age, sex and ethnicity; Model 2 was further adjusted for educational level, household income and employment status; Model 3 was additionally adjusted for body mass

index, smoking status, drinking status, physical activity, diet and antidepressant use based on Model 2; Model 4 was fully adjusted for all air pollutants based on Model 3. All P values were two-sided. CI confidence interval, HR hazard ratio, Ref reference.

Supplementary Table 18. Associations between environmental exposures at 300 m buffer and depression risk in prostate cancer patients (N=2,547).

Exposures	Model 1		Model 2		Model 3	
	HR (95% CI)	P	HR (95% CI)	P	HR (95% CI)	P
**Green space**						
Tertile 1	Ref		Ref		Ref	
Tertile 2	0.998 (0.650-1.533)	0.994	0.916 (0.595-1.410)	0.689	1.122 (0.721-1.745)	0.611
Tertile 3	0.752 (0.480-1.177)	0.213	0.759 (0.484-1.190)	0.229	0.961 (0.606-1.522)	0.864
P trend		0.207		0.227		0.861
Per 5% increment	0.981 (0.944-1.019)	0.328	0.985 (0.947-1.025)	0.467	1.005 (0.967-1.045)	0.782
**Blue space**						
Tertile 1	Ref		Ref		Ref	
Tertile 2	0.821 (0.536-1.257)	0.364	0.835 (0.545-1.280)	0.408	0.872 (0.565-1.345)	0.536
Tertile 3	0.760 (0.490-1.179)	0.221	0.782 (0.504-1.213)	0.272	1.061 (0.677-1.665)	0.795
P trend		0.217		0.267		0.848
Per 5% increment	0.991 (0.831-1.183)	0.924	1.010 (0.850-1.200)	0.911	1.063 (0.921-1.227)	0.403
**Natural environment**						
Tertile 1	Ref		Ref		Ref	
Tertile 2	0.881 (0.562-1.382)	0.582	0.882 (0.561-1.385)	0.585	0.820 (0.521-1.290)	0.391
Tertile 3	0.915 (0.593-1.411)	0.687	0.950 (0.615-1.468)	0.818	1.044 (0.673-1.618)	0.848
P trend		0.706		0.840		0.815
Per 5% increment	0.996 (0.962-1.031)	0.800	1.002 (0.967-1.038)	0.926	1.014 (0.979-1.051)	0.442

HRs, 95% CIs, and P values were estimated using Cox proportional hazards regression models: Model 1 was adjusted for age, sex and ethnicity; Model 2 was further adjusted for educational level, household income and employment status; Model 3 was fully adjusted for body mass index,

smoking status, drinking status, physical activity, diet and antidepressant use based on Model 2. All P values were two-sided. CI confidence interval, HR hazard ratio, Ref reference.

Supplementary Table 19. Associations between environmental exposures at 1000 m buffer and depression risk in prostate cancer patients (N=2,547).

Exposures	Model 1		Model 2		Model 3	
	HR (95% CI)	P	HR (95% CI)	P	HR (95% CI)	P
**Green space**						
Tertile 1	Ref		Ref		Ref	
Tertile 2	0.955 (0.621-1.468)	0.833	0.917 (0.595-1.414)	0.695	0.995 (0.640-1.545)	0.981
Tertile 3	0.713 (0.454-1.120)	0.142	0.716 (0.455-1.128)	0.150	0.832 (0.526-1.317)	0.432
P trend		0.136		0.145		0.427
Per 5% increment	0.965 (0.927-1.004)	0.074	0.966 (0.927-1.006)	0.095	0.977 (0.939-1.017)	0.261
**Blue space**						
Tertile 1	Ref		Ref		Ref	
Tertile 2	1.085 (0.706-1.669)	0.709	1.122 (0.729-1.725)	0.602	1.215 (0.783-1.885)	0.386
Tertile 3	0.927 (0.596-1.441)	0.736	0.934 (0.600-1.452)	0.761	1.129 (0.720-1.770)	0.596
P trend		0.741		0.768		0.578
Per 5% increment	1.015 (0.872-1.181)	0.849	1.026 (0.884-1.192)	0.733	1.067 (0.937-1.216)	0.327
**Natural environment**						
Tertile 1	Ref		Ref		Ref	
Tertile 2	0.701 (0.448-1.095)	0.118	0.699 (0.446-1.094)	0.117	0.807 (0.514-1.266)	0.350
Tertile 3	0.734 (0.479-1.126)	0.156	0.761 (0.495-1.169)	0.212	0.809 (0.524-1.248)	0.337
P trend		0.173		0.234		0.347
Per 5% increment	0.969 (0.935-1.005)	0.091	0.973 (0.938-1.010)	0.149	0.984 (0.949-1.021)	0.386

HRs, 95% CIs, and P values were estimated using Cox proportional hazards regression models: Model 1 was adjusted for age, sex and ethnicity; Model 2 was further adjusted for educational level, household income and employment status; Model 3 was fully adjusted for body mass index,

smoking status, drinking status, physical activity, diet and antidepressant use based on Model 2. All P values were two-sided. CI confidence interval, HR hazard ratio, Ref reference.

Supplementary Table 20. Associations between environmental exposures at 300 m buffer and depression risk in colorectal cancer patients (N=1,897).

Exposures	Model 1		Model 2		Model 3	
	**HR (95% CI)**	**P**	**HR (95% CI)**	**P**	**HR (95% CI)**	**P**
**Green space**						
Tertile 1	Ref		Ref		Ref	
Tertile 2	1.178 (0.777-1.785)	0.440	1.120 (0.738-1.700)	0.595	1.222 (0.798-1.871)	0.356
Tertile 3	0.804 (0.517-1.250)	0.333	0.836 (0.537-1.302)	0.427	0.944 (0.596-1.493)	0.804
P trend		0.295		0.399		0.767
Per 5% increment	0.983 (0.948-1.019)	0.348	0.991 (0.955-1.028)	0.622	0.997 (0.960-1.036)	0.879
**Blue space**						
Tertile 1	Ref		Ref		Ref	
Tertile 2	1.369 (0.900-2.082)	0.142	1.409 (0.926-2.144)	0.110	1.459 (0.95-2.24)	0.084
Tertile 3	0.962 (0.618-1.497)	0.862	1.029 (0.660-1.603)	0.901	1.111 (0.709-1.742)	0.645
P trend		0.799		0.951		0.689
Per 5% increment	0.751 (0.465-1.212)	0.241	0.819 (0.526-1.274)	0.375	0.845 (0.546-1.307)	0.449
**Natural environment**						
Tertile 1	Ref		Ref		Ref	
Tertile 2	0.794 (0.527-1.195)	0.269	0.785 (0.521-1.185)	0.249	1.000 (0.658-1.521)	1.000
Tertile 3	0.610 (0.401-0.928)	0.021	0.645 (0.422-0.985)	0.043	0.801 (0.518-1.239)	0.319
P trend		0.021		0.042		0.320
Per 5% increment	0.973 (0.939-1.008)	0.124	0.981 (0.946-1.017)	0.289	0.991 (0.957-1.027)	0.636

HRs, 95% CIs, and P values were estimated using Cox proportional hazards regression models: Model 1 was adjusted for age, sex and ethnicity; Model 2 was further adjusted for educational level, household income and employment status; Model 3 was fully adjusted for body mass index,

smoking status, drinking status, physical activity, diet and antidepressant use based on Model 2. All P values were two-sided. CI confidence interval, HR hazard ratio, Ref reference.

Supplementary Table 21. Associations between environmental exposures at 1000 m buffer and depression risk in colorectal cancer patients (N=1,897).

**Exposures**	**Model 1**		**Model 2**		**Model 3**	
	**HR (95% CI)**	**P**	**HR (95% CI)**	**P**	**HR (95% CI)**	**P**
**Green space**						
Tertile 1	Ref		Ref		Ref	
Tertile 2	0.644 (0.408-1.015)	0.058	0.639 (0.404-1.011)	0.056	0.709 (0.444-1.131)	0.149
Tertile 3	0.934 (0.629-1.387)	0.736	0.982 (0.658-1.465)	0.928	1.002 (0.666-1.508)	0.991
P trend		0.851		0.944		0.887
Per 5% increment	0.976 (0.940-1.014)	0.210	0.983 (0.946-1.022)	0.380	0.989 (0.951-1.028)	0.568
**Blue space**						
Tertile 1	Ref		Ref		Ref	
Tertile 2	0.725 (0.465-1.130)	0.155	0.740 (0.474-1.154)	0.184	0.751 (0.477-1.181)	0.215
Tertile 3	1.152 (0.774-1.716)	0.485	1.196 (0.803-1.783)	0.378	1.322 (0.880-1.985)	0.179
P trend		0.437		0.342		0.164
Per 5% increment	0.997 (0.873-1.139)	0.968	1.012 (0.885-1.156)	0.863	1.036 (0.904-1.187)	0.614
**Natural environment**						
Tertile 1	Ref		Ref		Ref	
Tertile 2	0.589 (0.379-0.916)	0.019	0.572 (0.366-0.892)	0.014	0.668 (0.425-1.049)	0.080
Tertile 3	0.765 (0.515-1.138)	0.187	0.798 (0.534-1.194)	0.273	0.895 (0.594-1.347)	0.594
P trend		0.228		0.336		0.656
Per 5% increment	0.976 (0.943-1.010)	0.157	0.982 (0.949-1.018)	0.323	0.992 (0.958-1.027)	0.651

HRs, 95% CIs, and P values were estimated using Cox proportional hazards regression models: Model 1 was adjusted for age, sex and ethnicity; Model 2 was further adjusted for educational level, household income and employment status; Model 3 was fully adjusted for body mass index,

smoking status, drinking status, physical activity, diet and antidepressant use based on Model 2. All P values were two-sided. CI confidence interval, HR hazard ratio, Ref reference.

Supplementary Table 22. Associations between environmental exposures at 300 m buffer and risk of depression after adjusting separately for air pollutants (N=21,507).

Exposures	Original *		Adjusted for NO_2		Adjusted for NO_x		Adjusted for PM_{10}		Adjusted for $PM_{2.5}$	
	HR (95% CI)	P	HR (95% CI)	P	HR (95% CI)	P	HR (95% CI)	P	HR (95% CI)	P
**Green space**										
Tertile 1	Ref		Ref		Ref		Ref		Ref	
Tertile 2	0.971 (0.856-1.101)	0.646	0.995 (0.874-1.132)	0.937	0.988 (0.870-1.123)	0.856	0.968 (0.853-1.098)	0.608	0.988 (0.869-1.124)	0.859
Tertile 3	0.902 (0.791-1.029)	0.126	0.962 (0.825-1.121)	0.621	0.946 (0.819-1.092)	0.448	0.891 (0.777-1.022)	0.099	0.951 (0.817-1.108)	0.523
P trend		0.127		0.631		0.454		0.101		0.533
Per 5% increment	0.992 (0.980-1.004)	0.192	1.000 (0.985-1.014)	0.955	0.997 (0.984-1.011)	0.688	0.991 (0.978-1.003)	0.143	0.998 (0.984-1.013)	0.838
**Blue space**										
Tertile 1	Ref		Ref		Ref		Ref		Ref	
Tertile 2	0.987 (0.871-1.119)	0.840	1.015 (0.893-1.155)	0.818	1.008 (0.888-1.145)	0.898	0.985 (0.869-1.117)	0.812	1.009 (0.888-1.148)	0.887
Tertile 3	0.867 (0.763-0.986)	0.030	0.893 (0.783-1.020)	0.095	0.886 (0.778-1.010)	0.069	0.865 (0.760-0.984)	0.028	0.891 (0.780-1.018)	0.090
P trend		0.031		0.091		0.068		0.028		0.087
Per 5% increment	0.966 (0.869-1.073)	0.519	0.974 (0.876-1.081)	0.616	0.972 (0.874-1.079)	0.592	0.965 (0.868-1.073)	0.513	0.975 (0.878-1.083)	0.634
**Natural environment**										
Tertile 1	Ref		Ref		Ref		Ref		Ref	
Tertile 2	0.940 (0.829-1.066)	0.333	0.967 (0.849-1.102)	0.614	0.961 (0.845-1.092)	0.540	0.936 (0.825-1.062)	0.305	0.961 (0.844-1.094)	0.545
Tertile 3	0.903 (0.792-1.028)	0.124	0.966 (0.828-1.126)	0.657	0.952 (0.822-1.102)	0.509	0.890 (0.776-1.020)	0.094	0.957 (0.820-1.118)	0.579
P trend		0.123		0.646		0.501		0.092		0.563
Per 5% increment	0.992 (0.982-1.003)	0.176	0.999 (0.986-1.012)	0.885	0.997 (0.985-1.010)	0.679	0.991 (0.979-1.002)	0.121	0.998 (0.985-1.012)	0.803

* HRs, 95% CIs, and P values were estimated using Cox proportional hazards regression model adjusted by age, sex, ethnicity, educational level, household income, employment status, body mass index, smoking status, drinking status, physical activity, diet and antidepressant use. All P values were two-sided. CI confidence interval, HR hazard ratio, NO_x nitrogen oxides, NO_2 nitrogen dioxide, $PM_{2.5}$ particulate matter with aerodynamic diameter < 2.5 μm, PM_{10} particulate matter with aerodynamic diameter <10 μm, Ref reference.

Supplementary Table 23. Associations between environmental exposures at 1000 m buffer and risk of depression after adjusting separately for air pollutants (N=21,507).

Exposures	Original *		Adjusted for NO_2		Adjusted for NO_x		Adjusted for PM_{10}		Adjusted for $PM_{2.5}$	
	HR (95% CI)	P	HR (95% CI)	P	HR (95% CI)	P	HR (95% CI)	P	HR (95% CI)	P
**Green space**										
Tertile 1	Ref		Ref		Ref		Ref		Ref	
Tertile 2	0.878 (0.774-0.997)	0.044	0.895 (0.781-1.025)	0.108	0.893 (0.784-1.017)	0.087	0.875 (0.771-0.993)	0.039	0.889 (0.781-1.014)	0.079
Tertile 3	0.842 (0.739-0.960)	0.010	0.877 (0.741-1.038)	0.127	0.872 (0.753-1.010)	0.068	0.830 (0.725-0.950)	0.007	0.870 (0.745-1.015)	0.077
P trend		0.010		0.121		0.067		0.007		0.070
Per 5% increment	0.983 (0.971-0.995)	0.006	0.985 (0.969-1.002)	0.075	0.986 (0.972-1.000)	0.043	0.981 (0.968-0.993)	0.003	0.985 (0.970-1.000)	0.043
**Blue space**										
Tertile 1	Ref		Ref		Ref		Ref		Ref	
Tertile 2	0.879 (0.774-0.997)	0.045	0.893 (0.786-1.014)	0.081	0.888 (0.782-1.008)	0.066	0.878 (0.773-0.996)	0.043	0.889 (0.783-1.010)	0.070
Tertile 3	0.896 (0.789-1.017)	0.088	0.904 (0.796-1.026)	0.119	0.898 (0.791-1.019)	0.096	0.895 (0.789-1.016)	0.087	0.902 (0.794-1.024)	0.110
P trend		0.085		0.117		0.094		0.084		0.108
Per 5% increment	0.995 (0.938-1.055)	0.868	0.998 (0.941-1.059)	0.958	0.996 (0.939-1.057)	0.893	0.995 (0.938-1.055)	0.868	0.997 (0.940-1.058)	0.933
**Natural environment**										
Tertile 1	Ref		Ref		Ref		Ref		Ref	
Tertile 2	0.814 (0.717-0.923)	0.001	0.820 (0.715-0.940)	0.005	0.824 (0.723-0.939)	0.004	0.810 (0.713-0.919)	0.001	0.820 (0.719-0.935)	0.003
Tertile 3	0.818 (0.719-0.930)	0.002	0.831 (0.700-0.986)	0.034	0.839 (0.724-0.973)	0.020	0.803 (0.701-0.919)	0.001	0.834 (0.713-0.976)	0.024
P trend		0.002		0.029		0.018		0.001		0.017
Per 5% increment	0.983 (0.973-0.994)	0.002	0.983 (0.968-0.999)	0.034	0.985 (0.973-0.998)	0.022	0.981 (0.970-0.992)	0.001	0.984 (0.970-0.997)	0.020

* HR and 95% CI were estimated by Cox regression model adjusted by age, sex, ethnicity, educational level, household income, employment status, body mass index, smoking status, drinking status, physical activity, diet and antidepressant use. All P values were two-sided. CI confidence interval, HR hazard ratio, NO_x nitrogen oxides, NO_2 nitrogen dioxide, $PM_{2.5}$ particulate matter with aerodynamic diameter < 2.5 μm, PM_{10} particulate matter with aerodynamic diameter <10 μm, Ref reference.

Supplementary Table 24. Associations between environmental exposures at 300 m and 1000 m buffers and risk of depression after adjusting separately for all air pollutants and air pollution score (N=21,507).

Exposures	300 m buffer				1000 m buffer			
	Adjusted for all air pollutants		Adjusted for air pollution score		Adjusted for all air pollutants		Adjusted for air pollution score	
	HR (95% CI)	P	HR (95% CI)	P	HR (95% CI)	P	HR (95% CI)	P
**Green space**								
Tertile 1	Ref		Ref		Ref		Ref	
Tertile 2	0.995 (0.874-1.133)	0.944	0.993 (0.873-1.129)	0.915	0.889 (0.772-1.023)	0.099	0.894 (0.783-1.021)	0.099
Tertile 3	0.958 (0.817-1.124)	0.598	0.960 (0.825-1.117)	0.597	0.857 (0.712-1.030)	0.100	0.878 (0.749-1.029)	0.108
P trend		0.613		0.606		0.114		0.108
Per 5% increment	0.999 (0.983-1.014)	0.872	0.999 (0.985-1.014)	0.924	0.980 (0.962-0.999)	0.035	0.986 (0.970-1.001)	0.066
**Blue space**								
Tertile 1	Ref		Ref		Ref		Ref	
Tertile 2	1.016 (0.892-1.156)	0.813	1.014 (0.892-1.152)	0.836	0.891 (0.784-1.013)	0.077	0.891 (0.785-1.012)	0.076
Tertile 3	0.893 (0.781-1.021)	0.098	0.893 (0.782-1.019)	0.093	0.902 (0.794-1.025)	0.114	0.902 (0.795-1.024)	0.110
P trend		0.094		0.090		0.114		0.108
Per 5% increment	0.974 (0.877-1.082)	0.621	0.974 (0.877-1.082)	0.624	0.999 (0.941-1.060)	0.976	0.997 (0.940-1.058)	0.933
**Natural environment**								
Tertile 1	Ref		Ref		Ref		Ref	
Tertile 2	0.969 (0.850-1.105)	0.640	0.966 (0.848-1.100)	0.601	0.810 (0.703-0.933)	0.003	0.823 (0.720-0.941)	0.004
Tertile 3	0.962 (0.820-1.128)	0.632	0.966 (0.828-1.127)	0.661	0.804 (0.667-0.969)	0.022	0.839 (0.714-0.986)	0.033
P trend		0.620		0.647		0.017		0.026
Per 5% increment	0.998 (0.984-1.012)	0.763	0.999 (0.986-1.012)	0.889	0.978 (0.961-0.995)	0.011	0.984 (0.971-0.999)	0.032

HR and 95% CI were estimated by Cox regression based on Model 3, adjusted for age, sex, ethnicity, educational level, household income, employment status, body mass index, smoking status, drinking status, physical activity, diet and antidepressant use, with additionally adjusted for all air pollutants or APS. All P values were two-sided. APS air pollution score, CI confidence interval, HR hazard ratio, Ref, reference.

Supplementary Table 25. Associations between environmental exposures at 300 m and 1000 m buffers and risk of depression after adjusting separately for cancer type and sleep pattern (N=21,507).

Exposures	300 m buffer				1000 m buffer			
	Adjusted for cancer type		Adjusted by sleep pattern		Adjusted for cancer type		Adjusted by sleep pattern	
	HR (95% CI)	P	HR (95% CI)	P	HR (95% CI)	P	HR (95% CI)	P
**Green space**								
Tertile 1	Ref		Ref		Ref		Ref	
Tertile 2	0.971 (0.856-1.101)	0.646	0.972 (0.857-1.103)	0.662	0.884 (0.779-1.003)	0.055	0.881 (0.776-1.000)	0.049
Tertile 3	0.902 (0.791-1.029)	0.126	0.904 (0.792-1.031)	0.132	0.843 (0.740-0.961)	0.010	0.842 (0.739-0.960)	0.010
P trend		0.141		0.133		0.010		0.010
Per 5% increment	0.992 (0.981-1.004)	0.205	0.992 (0.981-1.004)	0.202	0.983 (0.971-0.995)	0.006	0.983 (0.971-0.995)	0.006
**Blue space**								
Tertile 1	Ref		Ref		Ref		Ref	
Tertile 2	0.990 (0.874-1.123)	0.879	0.981 (0.865-1.112)	0.762	0.878 (0.773-0.996)	0.043	0.875 (0.771-0.993)	0.039
Tertile 3	0.866 (0.762-0.985)	0.029	0.865 (0.761-0.984)	0.027	0.892 (0.786-1.013)	0.077	0.888 (0.783-1.008)	0.067
P trend		0.029		0.028		0.075		0.065
Per 5% increment	0.962 (0.865-1.071)	0.482	0.964 (0.867-1.071)	0.492	0.992 (0.935-1.052)	0.783	0.994 (0.936-1.054)	0.834
**Natural environment**								
Tertile 1	Ref		Ref		Ref		Ref	
Tertile 2	0.939 (0.828-1.064)	0.323	0.941 (0.830-1.067)	0.341	0.817 (0.720-0.927)	0.002	0.814 (0.718-0.924)	0.001
Tertile 3	0.905 (0.794-1.031)	0.132	0.903 (0.793-1.029)	0.125	0.819 (0.719-0.932)	0.002	0.817 (0.718-0.929)	0.002
P trend		0.131		0.124		0.002		0.002
Per 5% increment	0.993 (0.982-1.003)	0.180	0.993 (0.982-1.004)	0.187	1.008 (0.992-1.024)	0.320	0.983 (0.973-0.994)	0.003

HR and 95% CI were estimated by Cox regression based on Model 3, adjusted for age, sex, ethnicity, educational level, household income, employment status, body mass index, smoking status, drinking status, physical activity, diet and antidepressant use. All P values were two-sided. CI confidence interval, HR hazard ratio, Ref reference.

Supplementary Table 26. Associations between environmental exposures at 300 m buffer and the risk of depression after removing antidepressant use from the covariate set (N=21,507), excluding those diagnosed with depression within 1 year (N=21,437) or 3 years (N=21,279) post-cancer, and those who died within 10 years after cancer diagnosis (N=20,522).

Exposures	Removing antidepressant use from the covariate set		Excluding depression cases within 1 year post-cancer		Excluding depression cases within 3 years post-cancer		Excluding death cases within 10 years post-cancer	
	**HR (95% CI)**	**P**	**HR (95% CI)**	**P**	**HR (95% CI)**	**P**	**HR (95% CI)**	**P**
**Green space**								
Tertile 1	Ref		Ref		Ref		Ref	
Tertile 2	1.015 (0.895-1.151)	0.814	0.989 (0.868-1.125)	0.862	0.977 (0.851-1.121)	0.739	0.980 (0.862-1.115)	0.763
Tertile 3	0.896 (0.786-1.022)	0.102	0.929 (0.811-1.063)	0.283	0.931 (0.807-1.074)	0.326	0.916 (0.801-1.048)	0.202
P trend		0.103		0.283		0.326		0.203
Per 5% increment	0.991 (0.979-1.003)	0.123	0.994 (0.982-1.006)	0.338	0.994 (0.981-1.007)	0.335	0.993 (0.981-1.006)	0.290
**Blue space**								
Tertile 1	Ref		Ref		Ref		Ref	
Tertile 2	0.980 (0.865-1.111)	0.754	0.990 (0.870-1.126)	0.877	0.969 (0.845-1.111)	0.653	0.985 (0.867-1.119)	0.819
Tertile 3	0.881 (0.775-1.002)	0.053	0.877 (0.769-1.000)	0.051	0.872 (0.758-1.002)	0.054	0.852 (0.747-0.972)	0.017
P trend		0.054		0.052		0.054		0.018
Per 5% increment	0.946 (0.857-1.044)	0.267	0.981 (0.884-1.087)	0.710	0.939 (0.830-1.061)	0.313	0.972 (0.874-1.080)	0.596
**Natural environment**								
Tertile 1	Ref		Ref		Ref		Ref	
Tertile 2	0.980 (0.864-1.111)	0.755	0.942 (0.828-1.072)	0.364	0.922 (0.804-1.058)	0.250	0.931 (0.819-1.059)	0.280
Tertile 3	0.893 (0.784-1.017)	0.088	0.920 (0.805-1.052)	0.222	0.914 (0.793-1.052)	0.210	0.907 (0.794-1.036)	0.151
P trend		0.088		0.221		0.208		0.150
Per 5% increment	0.991 (0.980-1.002)	0.114	0.994 (0.983-1.005)	0.290	0.993 (0.981-1.005)	0.247	0.993 (0.982-1.004)	0.240

HR and 95% CI were estimated by Cox regression based on Model 3, adjusted for age, sex, ethnicity, educational level, household income,

employment status, body mass index, smoking status, drinking status, physical activity, diet and antidepressant use. All P values were two-sided. CI confidence interval, HR hazard ratio, Ref reference.

Supplementary Table 27. Associations between environmental exposures at 1000 m buffer and the risk of depression after removing antidepressant use from the covariate set (N=21,507), excluding those diagnosed with depression within 1 year (N=21,437) or 3 years (N=21,279) post-cancer, and those who died within 10 years after cancer diagnosis (N=20,522).

Exposures	Removing antidepressant use from the covariate set		Excluding depression cases within 1 year post-cancer		Excluding depression cases within 3 years post-cancer		Excluding death cases within 10 years post-cancer	
	HR (95% CI)	P	HR (95% CI)	P	HR (95% CI)	P	HR (95% CI)	P
**Green space**								
Tertile 1	Ref		Ref		Ref		Ref	
Tertile 2	0.910 (0.802-1.032)	0.142	0.895 (0.786-1.019)	0.094	0.882 (0.768-1.013)	0.076	0.886 (0.779-1.009)	0.068
Tertile 3	0.853 (0.749-0.972)	0.017	0.864 (0.756-0.988)	0.032	0.856 (0.742-0.987)	0.032	0.858 (0.750-0.980)	0.024
P trend		0.017		0.033		0.032		0.025
Per 5% increment	0.983 (0.972-0.995)	0.006	0.985 (0.973-0.997)	0.017	0.984 (0.971-0.997)	0.014	0.985 (0.973-0.998)	0.019
**Blue space**								
Tertile 1	Ref		Ref		Ref		Ref	
Tertile 2	0.932 (0.822-1.058)	0.277	0.901 (0.792-1.026)	0.114	0.913 (0.795-1.048)	0.195	0.880 (0.773-1.001)	0.051
Tertile 3	0.902 (0.795-1.024)	0.110	0.903 (0.793-1.028)	0.124	0.915 (0.797-1.051)	0.211	0.887 (0.779-1.009)	0.069
P trend		0.109		0.121		0.209		0.066
Per 5% increment	0.976 (0.920-1.035)	0.415	1.001 (0.943-1.062)	0.975	0.983 (0.920-1.051)	0.621	0.998 (0.940-1.059)	0.940
**Natural environment**								
Tertile 1	Ref		Ref		Ref		Ref	
Tertile 2	0.813 (0.717-0.923)	0.001	0.826 (0.726-0.941)	0.004	0.844 (0.735-0.969)	0.016	0.815 (0.716-0.927)	0.002
Tertile 3	0.797 (0.700-0.907)	0.001	0.833 (0.729-0.951)	0.007	0.831 (0.721-0.957)	0.010	0.823 (0.721-0.939)	0.004
P trend		0.001		0.007		0.010		0.004
Per 5% increment	0.982 (0.971-0.993)	0.001	0.985 (0.974-0.996)	0.007	0.984 (0.972-0.995)	0.006	0.985 (0.974-0.996)	0.006

HR and 95% CI were estimated by Cox regression based on Model 3, adjusted for age, sex, ethnicity, educational level, household income,

employment status, body mass index, smoking status, drinking status, physical activity, diet and antidepressant use. All P values were two-sided. CI confidence interval, HR hazard ratio, Ref reference.

Supplementary Table 28. Associations between environmental exposures at 300 m buffer and the risk of depression, restricted to individuals who had lived at their current address for more than 10 years before baseline (N=15,162).

Exposures	Model 1		Model 2		Model 3	
	HR (95% CI)	P	HR (95% CI)	P	HR (95% CI)	P
**Green space**						
Tertile 1	Ref		Ref		Ref	
Tertile 2	1.107 (0.948-1.294)	0.199	1.057 (0.904-1.235)	0.490	1.023 (0.875-1.197)	0.773
Tertile 3	0.885 (0.751-1.044)	0.146	0.894 (0.758-1.054)	0.183	0.922 (0.781-1.088)	0.337
P trend		0.152		0.189		0.344
Per 5% increment	0.986 (0.972-1.000)	0.054	0.989 (0.975-1.004)	0.147	0.992 (0.978-1.007)	0.324
**Blue space**						
Tertile 1	Ref		Ref		Ref	
Tertile 2	0.958 (0.820-1.118)	0.584	0.974 (0.834-1.138)	0.741	0.969 (0.829-1.132)	0.691
Tertile 3	0.843 (0.717-0.990)	0.037	0.859 (0.731-1.009)	0.064	0.850 (0.723-0.999)	0.049
P trend		0.039		0.067		0.051
Per 5% increment	0.939 (0.845-1.044)	0.246	0.947 (0.853-1.052)	0.309	0.941 (0.841-1.053)	0.292
**Natural environment**						
Tertile 1	Ref		Ref		Ref	
Tertile 2	1.060 (0.906-1.239)	0.468	1.044 (0.893-1.221)	0.590	1.008 (0.862-1.179)	0.917
Tertile 3	0.866 (0.735-1.020)	0.086	0.889 (0.754-1.047)	0.160	0.930 (0.789-1.096)	0.387
P trend		0.087		0.164		0.394
Per 5% increment	0.983 (0.969-0.996)	0.013	0.987 (0.973-1.001)	0.059	0.991 (0.977-1.004)	0.183

HR and 95% CI were estimated by Cox regression: Model 1 was adjusted for age, sex and ethnicity; Model 2 was further adjusted for educational level, household income and employment status; Model 3 was fully adjusted for body mass index, smoking status, drinking status, physical activity, diet and antidepressant use based on Model 2. All P values were two-sided. CI confidence interval, HR hazard ratio, Ref reference.

Supplementary Table 29. Associations between environmental exposures at the 1000 m buffer and the risk of depression, restricted to individuals who had lived at their current address for more than 10 years before baseline (N=15,162).

Exposures	Model 1		Model 2		Model 3	
	HR (95% CI)	P	HR (95% CI)	P	HR (95% CI)	P
**Green space**						
Tertile 1	Ref		Ref		Ref	
Tertile 2	0.955 (0.817-1.116)	0.563	0.921 (0.788-1.077)	0.304	0.890 (0.761-1.041)	0.146
Tertile 3	0.795 (0.674-0.936)	0.006	0.804 (0.682-0.947)	0.009	0.831 (0.704-0.981)	0.028
P trend		0.006		0.009		0.028
Per 5% increment	0.974 (0.959-0.988)	<0.001	0.975 (0.961-0.990)	0.001	0.980 (0.965-0.995)	0.010
**Blue space**						
Tertile 1	Ref		Ref		Ref	
Tertile 2	0.890 (0.761-1.041)	0.144	0.901 (0.770-1.054)	0.194	0.846 (0.723-0.990)	0.037
Tertile 3	0.861 (0.735-1.009)	0.065	0.871 (0.743-1.020)	0.087	0.853 (0.728-0.999)	0.049
P trend		0.062		0.084		0.045
Per 5% increment	0.985 (0.911-1.066)	0.713	0.990 (0.916-1.070)	0.800	0.992 (0.919-1.069)	0.826
**Natural environment**						
Tertile 1	Ref		Ref		Ref	
Tertile 2	0.782 (0.669-0.914)	0.002	0.763 (0.652-0.892)	0.001	0.779 (0.665-0.911)	0.002
Tertile 3	0.716 (0.609-0.841)	<0.001	0.734 (0.625-0.863)	<0.001	0.794 (0.675-0.934)	0.005
P trend		<0.001		<0.001		0.005
Per 5% increment	0.971 (0.959-0.985)	<0.001	0.974 (0.961-0.988)	<0.001	0.980 (0.967-0.994)	0.005

HR and 95% CI were estimated by Cox regression: Model 1 was adjusted for age, sex and ethnicity; Model 2 was further adjusted for educational level, household income and employment status; Model 3 was fully adjusted for body mass index, smoking status, drinking status, physical activity, diet and antidepressant use based on Model 2. All P values were two-sided. CI confidence interval, HR hazard ratio, Ref reference.

Supplementary references

1 Wurtz, P. *et al.* Quantitative Serum Nuclear Magnetic Resonance Metabolomics in Large-Scale Epidemiology: A Primer on -Omic Technologies. *Am J Epidemiol* **186**, 1084-1096, doi:10.1093/aje/kwx016 (2017).

2 Soininen, P., Kangas, A. J., Wurtz, P., Suna, T. & Ala-Korpela, M. Quantitative serum nuclear magnetic resonance metabolomics in cardiovascular epidemiology and genetics. *Circ Cardiovasc Genet* **8**, 192-206, doi:10.1161/CIRCGENETICS.114.000216 (2015).

3 Ritchie, S. C. *et al.* Quality control and removal of technical variation of NMR metabolic biomarker data in ~120,000 UK Biobank participants. *Sci Data* **10**, 64, doi:10.1038/s41597-023-01949-y (2023).
