# Supplementary material for: Residential green space, air pollution, and related metabolites in association with depression among cancer survivors
Source: Nat Commun. 2026 Mar 9;17:3690. doi: 10.1038/s41467-026-70393-4 (PMC13100056; doi:10.1038/s41467-026-70393-4)
Supplement: Supplementary file 2 — Description of Additional Supplementary Files [file 41467_2026_70393_MOESM2_ESM.pdf]

Description of Additional Supplementary Files

Title: Supplementary Data 1

Description: List of nuclear magnetic resonance-based metabolite profiles. A total of 249 nuclear magnetic resonance-detected metabolites were included.

Title: Supplementary Data 2

Description: Sensitivity analyses of the associations between air pollution and depression risk among cancer survivors (N=21,507). * HRs, 95% CIs, and P values were estimated using Cox proportional hazards regression based on Model 3 adjusted for age, sex, ethnicity, educational level, household income, employment status, body mass index, smoking status, drinking status, physical activity, diet and antidepressant use, with additional adjustment for other corresponding confounders. All P values were two-sided. CI confidence interval, HR hazard ratio, Ref reference, HR hazard ratio, NO_x nitrogen oxides, NO_2 nitrogen dioxide, $PM_{2.5}$ particulate matter with aerodynamic diameter < 2.5 μm, PM_{10} particulate matter with aerodynamic diameter <10 μm.

Title: Supplementary Data 3

Description: Definitions and coding of variables in the UK Biobank.
