# Supplementary material for: Residential green space, air pollution, and related metabolites in association with depression among cancer survivors
Source: Nat Commun. 2026 Mar 9;17:3690. doi: 10.1038/s41467-026-70393-4 (PMC13100056; doi:10.1038/s41467-026-70393-4)
Supplement: Supplementary file 6 — Reporting Summary [file 41467_2026_70393_MOESM6_ESM.pdf]

nature portfolio

Corresponding author(s): Xue Li

Last updated by author(s): November 23, 2025

Reporting Summary

Nature Portfolio wishes to improve the reproducibility of the work that we publish. This form provides structure for consistency and transparency in reporting. For further information on Nature Portfolio policies, see our Editorial Policies and the Editorial Policy Checklist.

Statistics

For all statistical analyses, confirm that the following items are present in the figure legend, table legend, main text, or Methods section.

n/a	Confirmed	
☐	☒	The exact sample size (*n*) for each experimental group/condition, given as a discrete number and unit of measurement
☐	☒	A statement on whether measurements were taken from distinct samples or whether the same sample was measured repeatedly
☐	☒	The statistical test(s) used AND whether they are one- or two-sided<br>*Only common tests should be described solely by name; describe more complex techniques in the Methods section.*
☐	☒	A description of all covariates tested
☐	☒	A description of any assumptions or corrections, such as tests of normality and adjustment for multiple comparisons
☐	☒	A full description of the statistical parameters including central tendency (e.g. means) or other basic estimates (e.g. regression coefficient) AND variation (e.g. standard deviation) or associated estimates of uncertainty (e.g. confidence intervals)
☐	☒	For null hypothesis testing, the test statistic (e.g. *F*, *t*, *r*) with confidence intervals, effect sizes, degrees of freedom and *P* value noted<br>*Give P values as exact values whenever suitable.*
☒	☐	For Bayesian analysis, information on the choice of priors and Markov chain Monte Carlo settings
☒	☐	For hierarchical and complex designs, identification of the appropriate level for tests and full reporting of outcomes
☐	☒	Estimates of effect sizes (e.g. Cohen's *d*, Pearson's *r*), indicating how they were calculated

Our web collection on statistics for biologists contains articles on many of the points above.

Software and code

Policy information about availability of computer code

Data collection	Touch-screen and online questionnaires,physical measurements,and clinical laboratory test were used to collect data. The dataset analysed during this study is available in the UK Biobank (https://www.ukbiobank.ac.uk/) under application number 724597. Raw data from the UK Biobank cannot be shared per our Material Transfer Agreement; Access can be obtained by data application for the UK Biobank platform.
Data analysis	All analyses were conducted using R software (version 4.2.1). The analysis code is publicly available on GitHub (https://github.com/garic019/NCOMMS-25-08485-zjh) and has also been archived on Zenodo (https://zenodo.org/records/17684180).

For manuscripts utilizing custom algorithms or software that are central to the research but not yet described in published literature, software must be made available to editors and reviewers. We strongly encourage code deposition in a community repository (e.g. GitHub). See the Nature Portfolio guidelines for submitting code & software for further information.

Data

Policy information about availability of data

All manuscripts must include a data availability statement. This statement should provide the following information, where applicable:

- Accession codes, unique identifiers, or web links for publicly available datasets
- A description of any restrictions on data availability
- For clinical datasets or third party data, please ensure that the statement adheres to our policy

The UK Biobank patient-level data are available under restricted access for bona fide researchers; access can be obtained by applying at http://ukbiobank.ac.uk/

register-apply/. Raw data are protected and are not available due to data privacy laws. All participants provided informed written consent to take part in the study. Ethics approval for the UK Biobank was granted by the North West Multi-Centre Research Ethics Committee in 2006 and was updated regularly after that (https://www.ukbiobank.ac.uk/learn-more-about-uk-biobank/about-us/ethics). This study was conducted after approval by the UK Biobank under application reference 724597.

Research involving human participants, their data, or biological material

Policy information about studies with human participants or human data. See also policy information about sex, gender (identity/presentation), and sexual orientation and race, ethnicity and racism.

Reporting on sex and gender	In this study, only the term sex was utilized; the study design did not incorporate considerations or comparisons between sex and gender. Within the UK Biobank, sex was initially obtained from the central registry at the time of recruitment, with updates permitted by participants in certain instances. Notably, the UK Biobank does not provide disaggregated data distinguishing sex from gender. A total of 21,507 cancer survivors were included in the analysis, comprising 13,414 females (62.4%) and 8,093 males (37.6%). Subgroup analyses were conducted based on sex, and the corresponding results have been reported accordingly.
Reporting on race, ethnicity, or other socially relevant groupings	This study included ethnicity as a covariate, which was not used as a proxy for socioeconomic status. In addition, socially relevant groupings included educational level, employment status, and household income. We provided clear definitions and categorizations for these terms. For educational level, this was self-reported in response to the question: "Which of the following qualifications do you have?" Educational attainment was categorized into two groups: “College” (College or University degree) and “Below College” (including A levels/AS levels or equivalent, O levels/GCSEs or equivalent, CSEs or equivalent, NVQ, HND, HNC or equivalent, other professional qualifications such as nursing or teaching, and no qualifications). Employment status was based on the self-reported response to the question: "Which of the following describes your current situation? (You can select more than one answer)." We classified participants as either currently employed or currently unemployed. Household income was based on the self-reported response to the question: "What is the average total income before tax received by your household?" It was categorized into three groups: high (≥ £52,000), middle (£18,000–£51,999), and low (< £18,000). All of these variables were adjusted for as potential confounders in multivariable models (Model 2 and Model 3). Additionally, stratified analyses were conducted to assess the potential effect modification of these variables on the association between the exposure and the outcome.
Population characteristics	The study included 21,507 cancer survivors with a median age of 62 years. Among them, 13,414 (62.4%) were female and 8,093 (37.6%) were male. A total of 19,853 participants (92.3%) were White. Regarding educational attainment, 6,386 participants (29.7%) had a university or college degree. In terms of body mass index (BMI), 8,930 participants (41.5%) were classified as overweight, and 5,409 (25.1%) as obese. A total of 12,578 participants (58.5%) were currently employed. Household income was categorized as low for 6,599 participants (30.7%), middle for 11,133 (51.8%), and high for 3,775 (17.6%). Physical activity level was classified as high for 10,577 participants (49.2%) and low for 10,930 participants (50.8%). Additionally, 1,851 participants (8.6%) were current smokers, 19,670 (91.5%) were current alcohol consumers, and 7,881 (36.6%) were classified as having a healthy diet. Moreover, 1,621 participants (7.5%) reported current use of antidepressants.
Recruitment	Participants were recruited from the UK Biobank, a large-scale population-based cohort study comprising over 500,000 individuals aged 40–69 years at baseline (2006–2010), who were identified through National Health Service (NHS) patient registers and invited to participate. Participants voluntarily attended one of 22 assessment centers across the UK, where they completed questionnaires, interviews, physical measurements, and biological sample collection. As participation was voluntary, self-selection bias is possible. Individuals who enrolled in the UK Biobank may be healthier, more health-conscious, and from higher socioeconomic backgrounds than the general population. This "healthy volunteer" effect may limit the generalizability of findings to broader or more diverse populations.
Ethics oversight	The UK Biobank study was approved by the North West Multi-Centre Research Ethics Committee and all participants provided written informed consent (https://www.ukbiobank.ac.uk/learn-more-about-uk-biobank/about-us/ethics). The study protocol is available online (https://www.ukbiobank.ac.uk).

Note that full information on the approval of the study protocol must also be provided in the manuscript.

Field-specific reporting

Please select the one below that is the best fit for your research. If you are not sure, read the appropriate sections before making your selection.

☒ Life sciences ☐ Behavioural & social sciences ☐ Ecological, evolutionary & environmental sciences

For a reference copy of the document with all sections, see nature.com/documents/nr-reporting-summary-flat.pdf

Life sciences study design

All studies must disclose on these points even when the disclosure is negative.

Sample size	This study was based on the UK Biobank and included a total of 21,507 cancer survivors in the final analysis. Using a prospective cohort design, we investigated the associations of residential green space, air pollution, and related metabolites with depression risk among cancer survivors. As this is a cohort study, no formal sample size calculation was performed prior to the study. However, the inclusion of 21,507 cancer survivors from the UK Biobank provides a sufficiently large sample to detect meaningful associations. The large cohort size enhances statistical power, allows for robust adjustment of potential confounders, and enables subgroup analyses, thereby supporting the reliability and generalizability of our findings.

Data exclusions	We initially included 93,677 participants with a cancer diagnosis in the UK Biobank. We excluded individuals diagnosed with cancer after baseline (n = 65,195), those with less than five years of cancer survivorship (n = 714), participants with missing data on green space, blue space, and natural environment exposures (n = 3,393), those with a diagnosis of depression prior to baseline (n = 303), and those diagnosed with other psychiatric disorders (n = 2,565). After applying these exclusion criteria, a total of 21,507 cancer survivors were included in the final analysis.
Replication	This study is based on observational data from the UK Biobank and does not involve laboratory experiments. Reproducibility was ensured through predefined inclusion and exclusion criteria, standardized data preprocessing procedures, and the use of established statistical methods. All analytical workflows were executed consistently across replications, and all attempts at reproducing the results were successful.
Randomization	NA, this study is a perspective cohort study.
Blinding	NA, this study is a perspective cohort study.

Reporting for specific materials, systems and methods

We require information from authors about some types of materials, experimental systems and methods used in many studies. Here, indicate whether each material, system or method listed is relevant to your study. If you are not sure if a list item applies to your research, read the appropriate section before selecting a response.

Materials & experimental systems

n/a	Involved in the study	
☒	☐	Antibodies
☒	☐	Eukaryotic cell lines
☒	☐	Palaeontology and archaeology
☒	☐	Animals and other organisms
☒	☐	Clinical data
☒	☐	Dual use research of concern
☒	☐	Plants

Methods

n/a	Involved in the study	
☒	☐	ChIP-seq
☒	☐	Flow cytometry
☒	☐	MRI-based neuroimaging

Plants

Seed stocks	NA
Novel plant genotypes	NA
Authentication	NA
